# High Abundance of genus *Prevotella* in the gut of perinatally HIV-infected children is associated with IP-10 levels despite therapy

**DOI:** 10.1038/s41598-018-35877-4

**Published:** 2018-12-05

**Authors:** Urvinder S. Kaur, Anita Shet, Niharika Rajnala, Bindu Parachalil Gopalan, Preeti Moar, Himanshu D, Balendra Pratap Singh, Rupesh Chaturvedi, Ravi Tandon

**Affiliations:** 1 Laboratory of AIDS Research and Immunology, School of Biotechnology, Jawaharlal Nehru University, New Delhi, India; 2 International Vaccine Access Center, Johns Hopkins School of Public Health, Baltimore, USA; 3 Division of Infectious Diseases, St. John’s Research Institute, St. John’s National Academy of health Sciences, Bangalore, India; 4 Department of Medicine, King Georges Medical University, Lucknow, India; 5 Department of Prosthodontics, King Georges Medical University, Lucknow, India; 6 Host Pathogen Interaction Laboratory, School of Biotechnology, Jawaharlal Nehru University, New Delhi, India

## Abstract

Perinatal HIV infection is characterized by faster HIV disease progression and higher initial rate of HIV replication compared to adults. While antiretroviral therapy (ART) has greatly reduced HIV replication to undetectable levels, there is persistent elevated inflammation associated with HIV disease progression. Alteration of gut microbiota is associated with increased inflammation in chronic adult HIV infection. Here, we aim to study the gut microbiome and its role in inflammation in treated and untreated HIV-infected children. Examination of fecal microbiota revealed that perinatally infected children living with HIV had significantly higher levels of genus *Prevotella* that persisted despite ART. These children also had higher levels of soluble CD14 (sCD14), a marker of microbial translocation, and IP-10 despite therapy. The *Prevotella* positively correlated with IP-10 levels in both treated and untreated HIV-infected children, while genus *Prevotella* and species *Prevotella copri* was inversely associated with CD4 count. Relative abundance of genus *Prevotella* and species *Prevotella copri* showed positive correlation with sCD14 in ART-suppressed perinatally HIV-infected children. Our study suggests that gut microbiota may serve as one of the driving forces behind the persistent inflammation in children despite ART. Reshaping of microbiota using probiotics may be recommended as an adjunctive therapy along with ART.

## Introduction

Due to advent of antiretroviral therapy (ART), HIV infection has now become a chronic disease that can be controlled with the ongoing treatment^1^. In addition, there has been 70% reduction in perinatal HIV infection between 2000 and 2015 as a result of introduction of ART in HIV-infected pregnant women (Children and HIV, fact sheet, UNAIDS, 2016). While ART substantially reduces HIV viral replication in infected individuals, chronic complications such as cardiovascular disease, metabolic syndrome, liver disease, kidney disease, neurocognitive impairment, and accelerated aging are being reported in higher frequency among horizontally HIV-infected adults^2,3^. During HIV infection, substantial CD4+T cells depletion occurs in the gastrointestinal tract. This loss is preferentially within Th17 cells that help in mucosal defense and maintain the intestinal epithelial barrier^4^. The depletion of Th17 cells in intestine has been reported to be associated with the disruption of epithelial barrier in HIV-infected individuals, leading to translocation of microbial products such as bacterial DNA^5^ and lipopolysaccharides^6^ into systemic circulation. Microbial translocation leads to immune activation and inflammation^7^ that persists despite suppressive ART^8–11^ and may contribute to non-AIDS complications^12,13^.

Dysbiosis is associated with different inflammatory conditions like inflammatory bowel diseases (IBD)^14^, irritable bowel syndrome (IBS)^15^, diabetes^16^, obesity^17,18^, rheumatoid arthritis^19^, allergic disease^20^ and multiple sclerosis^21,22^. Alteration in intestinal microbiota has also been reported in HIV-infected individuals^23–34^. Gori *et al*. showed increased prevalence of *Candida albicans* and *Pseudomonas aeruginosa* with significantly lower abundance of beneficial bacteria like Lactobacilli and *Bifidobacteria* in the stool samples of treatment naïve adults with acute HIV infection compared to healthy controls^24^. During early HIV infection, high abundance of Lactobacillales in the gut of untreated HIV-infected subjects is associated with increased CD4+ cell percentage, lower viral load and reduced microbial translocation that continued even after ART^35^. Later, several authors showed gut microbiota compositional changes during chronic HIV infection. They found significant increase in the relative abundance of genus *Prevotella* and decreased *Bacteroides* in the mucosal and fecal samples of adult HIV-infected subjects^25–28,32,36^. Furthermore, HIV-associated taxa in adults strongly correlated with mucosal and systemic inflammation^25,27,31,33^.

Most of the previous work on the role of gut microbiota in HIV pathogenesis focused adult HIV-infected subjects. A recent study by Bender *et al*. reported the gut microbiome differences in HIV exposed uninfected infants (HEU) when compared to HIV unexposed uninfected children (HUU). This difference in the gut microbiome of HEU infants was associated with distinct breast milk oligosaccharides of HIV-infected mothers^37^. However, there are currently no reports on the role of gut microbiota in intestinal barrier integrity and microbial translocation in the setting of perinatal HIV-infection.

Children with HIV infection experience faster disease progression compared to adults, with death occurring among up to 50% of untreated children within 2 years of life^38^. This rapid progression is associated with high viral load and increased depletion of CD4+ lymphocytes in children than adults. Perinatally HIV-infected children also showed viral mutation and poor drug adherence^39^. Unlike adult HIV infection, perinatally HIV-infected children are exposed to HIV either in utero or during birth. This raises a possibility that perinatally HIV-infected children may either be tolerized and have least impact on gut microbiome or have distinct pattern of gut microbiota compared to HIV-infected adults. Given the fact that the development of gut microbiota starts at birth and there is striking differences in HIV pathogenesis between adult and vertical HIV infection, we sought to study the composition and role of gut microbiota among treatment naïve and ART-suppressed perinatally HIV-infected children. In this study, we identified the difference in the fecal microbiota as a substitute to gut microbiota of perinatally HIV-infected children and age-matched uninfected controls. We carried out high throughput 16S rRNA gene sequencing to assess fecal microbiota and explored the relationship of unique fecal microbiota composition of treatment naïve and ART-suppressed perinatally HIV-infected children with systemic inflammation and microbial translocation.

## Results

### Perinatally HIV-infected children show a distinct pattern of gut microbiota compared to uninfected controls

**Figure 1:**
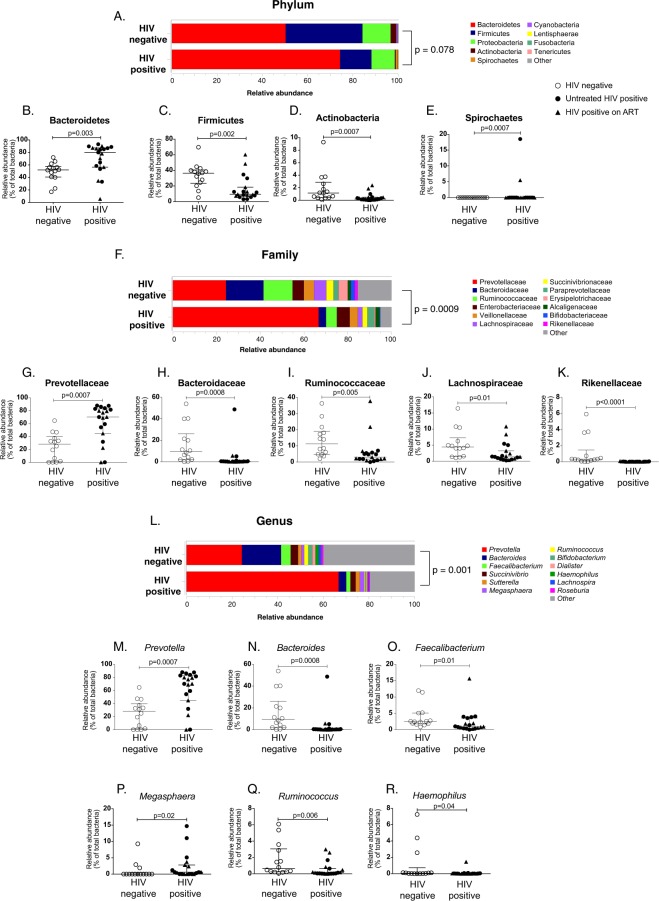
(**A**) Stacked bar plot depicting mean relative abundance of top nine abundant phyla in the fecal samples of HIV negative and HIV positive children. Dot Plots representing relative abundance of phyla (**B**) Bacteroidetes (**C**) Firmicutes, (**D**) Actinobacteria, and (**E**) Spirochaetes in HIV negative and HIV positive children. (**F**) Stacked bar plot showing mean relative abundance of top twelve abundant families in the fecal samples of HIV negative and HIV positive children. Dot Plots representing relative abundance of families (**G**) Prevotellaceae, (**H**) Bacteroidaceae (**I**) Ruminococcaceae (**J**) Lachnospiraceae and (**K**) Rikenellaceae in HIV negative and HIV positive children. (**L**) Stacked bar plot showing mean relative abundance of top twelve abundant genera in the fecal samples of HIV negative and HIV positive children. Dot Plots representing relative abundance of genera (**M**) *Prevotella* (**N**) *Bacteroides* (**O**) *Faecalibacterium* (**P**) *Megasphaera* (**Q**) *Ruminococcus* and (**R**) *Haemophilus* in HIV negative and HIV positive children. The horizontal line and deviation in the dot plot represents median and interquartile range (IQR), respectively. Mann-Whitney-U-test was performed for statistical analysis. p < 0.05 was considered to be significant. Significant differences in the overall relative abundance of fecal microbiota at phylum, family and genus level between HIV negative and HIV positive children were estimated using adonis function of vegan R package.

To determine the composition of gut microbiota in the setting of perinatal HIV infection, we assessed fecal microbiota in perinatally HIV-infected children. We determined the relative abundance of fecal microbiota at the phylum, family, and genus level and compared their levels between perinatally HIV-infected children and uninfected controls. Perinatally HIV-infected children showed distinct pattern of fecal microbiota compared to uninfected controls (Fig. 1A–R). The three most predominant phyla accounting for >97% of the overall bacterial abundance in fecal samples of both HIV-infected children and uninfected controls were Bacteroidetes, Firmicutes and Proteobacteria. No significant difference was observed in the overall relative abundances at the phylum level between perinatally HIV-infected children and uninfected controls (p = 0.08) (Fig. 1A). However, when relative abundance of individual phylum was compared between HIV-infected children and controls, Bacteroidetes and Spirochaetes were found to be significantly elevated (p = 0.002 and p = 0.0007, respectively) whereas Firmicutes, Actinobacteria and Cyanobacteria were significantly lower (p = 0.002, p = 0.0007 and p = 0.03, respectively) in HIV-infected children compared to uninfected controls (Figs 1B–E and S1B). The relative abundance of the remaining phyla did not show any significant difference between perinatally HIV-infected children and uninfected controls (Fig. S1A,C–E). On comparing the bacteria at the family and genus level, we observed overall relative abundances to be significantly different between HIV-infected children and uninfected controls (p = 0.0001 and p = 0.002, respectively) (Fig. 1F,L). Then, we individually compared the relative abundance of top abundant bacterial families between perinatally HIV-infected children and uninfected controls and observed that the relative abundance of Prevotellaceae was significantly higher (p = 0.0007), while Bacteroidaceae (p = 0.0008), Ruminococcaceae (p = 0.005), Lachnospiraceae (p = 0.01), and Rikenellaceae (p < 0.0001) were significantly lower in perinatally HIV-infected children compared to uninfected controls (Figs 1G–K, S2A–G). Among the abundant genera, the relative abundance of *Prevotella and Megasphaera* were significantly increased (p = 0.0007, and p = 0.02, respectively), whereas that of *Bacteroides* (p = 0.0008), *Faecalibacterium* (p = 0.01), *Ruminococcus* (p = 0.006) and *Haemophilus* (p = 0.04) was significantly lower in HIV-infected children compared to uninfected controls (Fig. 1M–R). The relative abundance of remaining abundant families and genera did not show difference between perinatally HIV-infected children and uninfected controls (Figs S2 and S3). The ratio of abundance of *Bacteroides* to *Prevotella* was significantly lower in perinatally HIV-infected children compared to uninfected controls (p = 0.0008, Fig. S5A). Our data shows that perinatally HIV-infected children have distinct pattern of gut microbiota compared to uninfected controls. The median values of relative abundance of significantly distinct phyla, families, and genera between perinatally HIV-infected children and uninfected controls are shown in Table S1.

### Impact of ART on the fecal microbiota in perinatally HIV-infected children

**Figure 2:**
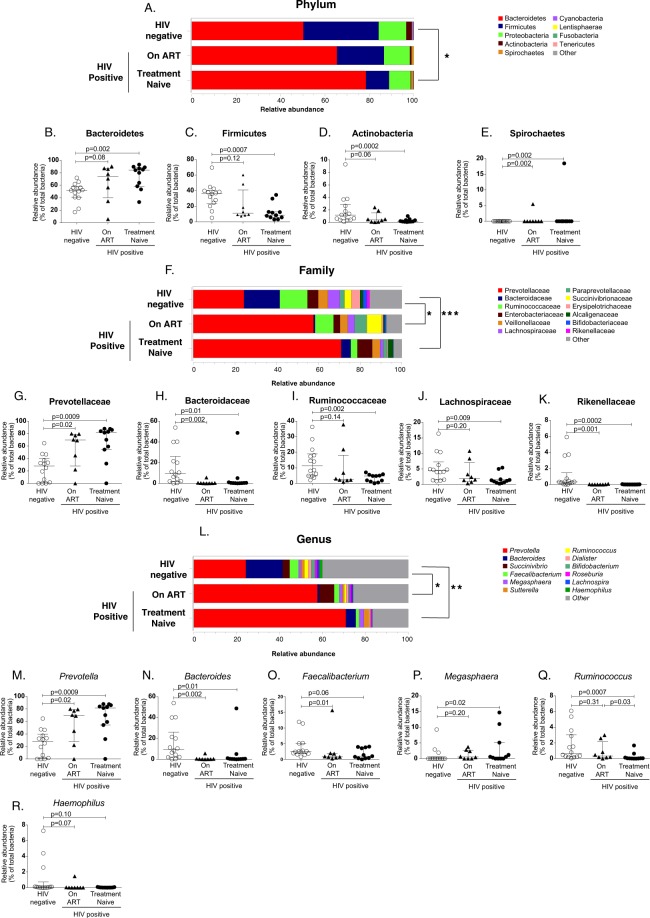
(**A**) Stacked bar plot depicting mean relative abundance of top nine abundant phyla n the fecal samples of HIV negative, on ART and treatment naïve perinatally HIV-infected children. Comparison of relative abundance of phyla (**B**) Bacteroidetes (**C**) Firmicutes, (**D**) Actinobacteria and (**E**) Spirochaetes across HIV negative, on ART and treatment naïve perinatally HIV-infected children. (**F**) Stacked bar plot showing mean relative abundance of top twelve abundant families in the fecal samples of HIV negative, on ART and treatment naïve perinatally HIV-infected children. Comparison of relative abundance of families (**G**) Prevotellaceae, (**H**) Bacteroidaceae (**I**) Ruminococcaceae (**J**) Lachnospiraceae and (**K**) Rikenellaceae across HIV negative, on ART and treatment naïve perinatally HIV-infected children. (**L**) Stacked bar plot showing mean relative abundance of top twelve abundant genera in the fecal samples of HIV negative, on ART and treatment naïve perinatally HIV-infected children. Comparison of relative abundance of genera (**M**) *Prevotella* (**N**) *Bacteroides* (**O**) *Faecalibacterium* (**P**) *Megasphaera* (**Q**) *Ruminococcus* and (**R**) *Haemophilus* across HIV negative, on ART and treatment naïve perinatally HIV-infected children. The horizontal line and deviation in the dot plot represents median and interquartile range (IQR), respectively. Kruskal-Wallis one-way ANOVA with Dunn’s multiple comparison was performed for statistical analysis. p < 0.05 was considered to be significant. Significant differences in the overall relative abundance of fecal microbiota at phylum, family and genus level between HIV negative controls, On ART and treatment naïve HIV-infected children were estimated using adonis function of vegan R package. *p < 0.05, **p < 0.005, p < 0.0005.

In order to determine the impact of antiretroviral therapy on fecal microbiota of perinatally HIV-infected children, we segregated perinatally HIV-infected children into treatment naïve and ART-experienced group and compared fecal microbiota across three groups. At the level of phylum, we observed overall relative abundance to be significantly different between treatment naïve HIV-infected children and uninfected controls (p = 0.01) but not between ART-suppressed HIV-infected children and uninfected controls (p = 0.27) (Fig. 2A). The relative abundance of Bacteroidetes (p = 0.002) and Spirochaetes (p = 0.002) were found to be significantly higher whereas Firmicutes (p = 0.0007) and Actinobacteria (p = 0.0002) were significantly lower in treatment naïve HIV-infected children compared to uninfected controls (Fig. 2B–E). In ART-suppressed HIV-infected children, the relative abundance of bacterial phyla were normalized to that of uninfected controls except Spirochaetes which remain elevated despite therapy (p = 0.002) (Fig. 2E). Comparisons of bacterial families showed significant difference in the overall relative abundances between uninfected controls and treatment naïve (p = 0.001) or ART-suppressed HIV-infected children (p = 0.02) (Fig. 2F). The relative abundance of Prevotellaceae were significantly increased (p = 0.0009 and p = 0.02 respectively) whereas Bacteroidaceae (p = 0.01 and p = 0.002) and Rikenellaceae (p = 0.0002) were significantly lower in both treatment naïve and ART-suppressed HIV-infected groups compared to uninfected controls (Fig. 2G,H). The relative abundance of other abundant families Ruminococcaceae (p = 0.002) and Lachnospiraceae (p = 0.009) were significantly lower in treatment naïve HIV-infected children whereas no significant difference was found in these families in ART-suppressed HIV-infected children compared to controls. (Fig. 2G–K). We also found overall relative abundance to be significantly different at the genus level between uninfected controls and treatment naïve (p = 0.005) or ART-suppressed HIV-infected children (p = 0.01) (Fig. 2L). ART did not show any effect on the relative abundance of *Prevotella* and *Bacteroides*. Compared to uninfected controls, treatment naïve and ART-suppressed HIV-infected children had significantly higher relative abundance of *Prevotella* (p = 0.0009 and p = 0.02 respectively) and lower abundance of *Bacteroides* (p = 0.01 and p = 0.002 respectively) (Fig. 2M,N). The relative abundance of *Megasphaera* was significantly higher (p = 0.02) and *Ruminococcus* (p = 0.0007) was significantly lower only in treatment naïve HIV-infected children compared to controls (Fig. 2P,Q). However, compared to treatment naïve HIV-infected children, the relative abundance of *Ruminococcus* was significantly higher in ART-suppressed children, suggesting the complete restoration of this genus by ART (Fig. 2Q). We did not observe difference in the relative abundance of *Faecalibacterium* (p = 0.06) and *Haemophilus* (p = 0.1) in treatment naïve HIV-infected children compared to controls (Fig. 2O,R). However, ART-suppressed HIV-infected children had decreased *Faecalibacterium* compared to controls (Fig. 2O). Our results suggest that the fecal microbiota of HIV-infected children did not completely get restored after ART treatment. The median values of relative abundance of significantly distinct phyla, families, and genera among treatment naïve, ART-suppressed HIV-infected children and uninfected controls are shown in Table S2.

### Microbial taxa associated with HIV and ART usage

**Figure 3:**
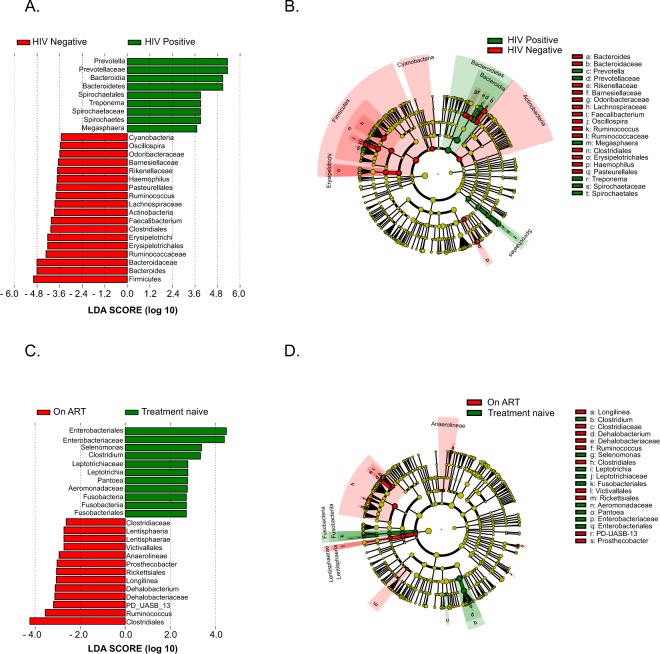
(**A**) LEfSe analysis showing the bacterial taxa that were significantly different in abundance between HIV positive children and HIV negative controls. Taxa enriched in HIV positive children are shown in green with positive LDA score and HIV negative controls in red with negative LDA score. Taxa passing LDA threshold value of >2.4 are only shown. (**B**) Cladogram representing the differentially abundant taxa between HIV positive children and HIV negative controls. Bacterial taxa enriched in HIV positive children and HIV negative controls are shown in green and red respectively. Brightness of each dot is proportional to taxon abundance. (**C**) LEfSe identifies significantly differentially abundant taxa between treatment naïve and On ART HIV-infected children and HIV. Taxa enriched in treatment naive HIV-infected children are shown in green with positive LDA score and On ART HIV-infected children in red with negative LDA score. Taxa passing LDA threshold value of >2.4 are only shown. (**D**) Taxonomic Cladogram. Red and green shows bacterial taxa enriched in On ART and treatment naïve HIV-infected children and uninfected controls, respectively. Brightness of each dot is proportional to taxon abundance.

In order to gain insight into the bacterial taxa mostly associated with HIV, we performed linear discriminant analysis effect size analysis (LEfSe) that revealed significant difference in the abundance of 27 taxa between perinatally HIV-infected children and uninfected controls (LDA score >2.5, Fig. 3A,B). In addition to the taxa shown in Fig. 1, LEfSe revealed decreased Odoribacteraceae, Barnesiellaceae and *Oscillospira* in HIV-infected children compared to controls (Fig. 3A,B). Further, we carried out LEfSe analysis to determine the taxa most associated with ART usage. We found 32 significant differentially abundant taxa between treatment naïve and ART-suppressed children. Of the families, Clostridiaceae and Dehalobacteriaceae were enriched whereas Enterobacteriaceae and Leptotrichiaceae were decreased in ART-suppressed HIV-infected children. Additionally, genera *Selenomonas*, *Clostridium*, *Leptotrichia* and *Pantoea* were enriched in treatment naïve HIV-infected children whereas *Longilinea*, *Dehalobacterium*, *PD-UASB-13* were more prevalent in ART-suppressed children (Fig. 3C,D).

### Analysis of diversity of microbial community

**Figure 4:**
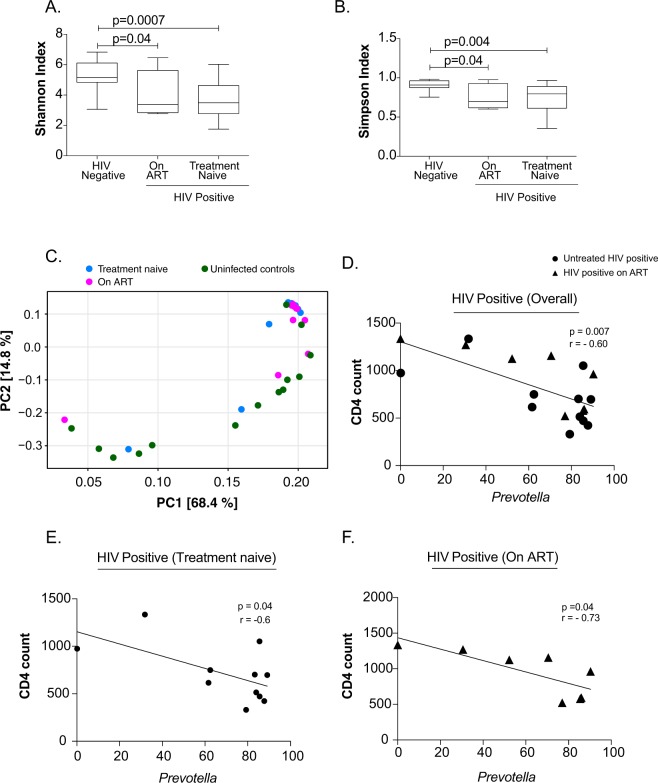
(**A**) Shannon and (**B**) Simpson indices were used to estimate the α-diversity of fecal microbiome of HIV negative controls, ART-suppressed and treatment naïve perinatally HIV infected children. (**C**) Plot of principle component analysis of fecal microbiome of HIV negative controls (green), On ART (pink) and treatment naïve (blue) perinatally HIV infected subjects. Association between relative abundance of *Prevotella* and CD4 count in (**D**) HIV positive children (overall), (**E**) treatment naïve and (**F**) On ART perinatally HIV infected children. Statistical analysis was carried out using the Spearman *t*-test. A linear regression line is included in each plot. p < 0.05 was considered to be significant.

Alpha diversity is the assessment of diversity within a habitat. We analysed the alpha diversity of fecal microbiota using the Shannon and Simpson index. The fecal microbiota diversity of both treatment-naïve and ART-suppressed HIV-infected children was significantly decreased when compared to uninfected controls (Fig. 4A,B). Beta diversity with principal component analysis (PCA) showed that the fecal microbiota composition of most of the treatment-naïve and ART-suppressed HIV-infected children were grouped into cluster and different from uninfected controls (Fig. 4C).

### *Prevotella* is indirectly associated with CD4 T cells in perinatally HIV-infected children

As CD4 depletion is a hallmark of HIV disease progression, we sought to determine association of genera that were significantly differentially abundant in HIV-infected children with CD4 count. The genus *Prevotella* was only found to be indirectly associated with CD4 count in all HIV-infected children (p = 0.007; r = −0.6) (Figs 4D and 6C). The indirect association between *Prevotella* and CD4 T cell count remained significant in treatment naïve and ART-suppressed perinatally HIV-infected children (Fig. 4E,F). *Prevotell*a did not show any correlation with CD4 T cell count in uninfected controls (data not shown).

### Increased abundance of *Prevotella copri* in the gut of perinatally HIV-infected children despite ART

**Figure 5:**
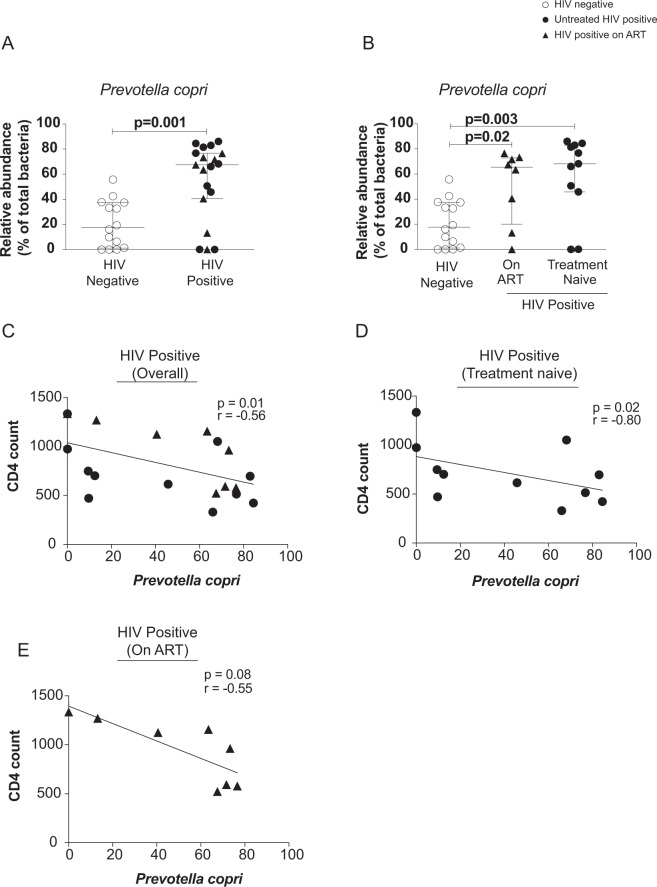
Dot plots representing relative abundance of *Prevotella copri* in the fecal sample of (**A**) HIV positive and HIV negative children and (**B**) HIV negative, On ART and treatment naïve perinatally HIV-infected children. The horizontal line and deviation in the dot plot represents median and interquartile range (IQR), respectively. Mann-Whitney *U-*test and Kruskal-Wallis one-way ANOVA with Dunn’s multiple comparison were performed for statistical analysis. The p < 0.05 was considered to be significant. Correlation between relative abundance of *Prevotella copri* and CD4 count in (**C**) HIV positive children (overall), (**D**) treatment naïve. (**E**) On ART perinatally HIV infected children. Statistical analysis was carried out using Spearman *t*-test. A linear regression line is included in each plot. p < 0.05 was considered to be significant.

We were intrigued by the increased relative abundance of *Prevotella* in perinatally HIV-infected children despite suppressive therapy and wanted to further characterize the genus *Prevotella* to species level. At species level, we identified *Prevotella copri and Prevotella stercorea*. However, only the relative abundance of *Prevotella copri* was found to be elevated in HIV-infected children compared to controls (p = 0.001) (Fig. 5A). The relative abundance of *Prevotella copri* was also significantly elevated in treatment naïve (p = 0.003) and ART-suppressed HIV-infected children (p = 0.02) compared to uninfected controls, suggesting that ART does not normalize the levels of *Prevotella copri* in HIV-infected children (Fig. 5B). Additionally, the relative abundance of other abundant species *Bacteroides plebieus*, *Bacteroides uniformis and Faecalibacterium prausnitzii* in the fecal sample of HIV-infected children was significantly decreased compared to controls (Fig. S4) and remained lowered regardless of treatment. Like genus *Prevotella*, the relative abundance of *P*. *copri* also showed indirect association with CD4 T cells in overall and treatment naïve perinatally HIV-infected children (Fig. 5C,D) however it failed to achieve level of significance in ART-suppressed HIV-infected children (p = 0.08; Fig. 5E). The median values for the relative abundance of *Prevotella copri* in three groups are shown in Table S2.

### Soluble IP-10 is elevated in HIV-infected children and are associated with *Prevotella* despite ART

**Figure 6:**
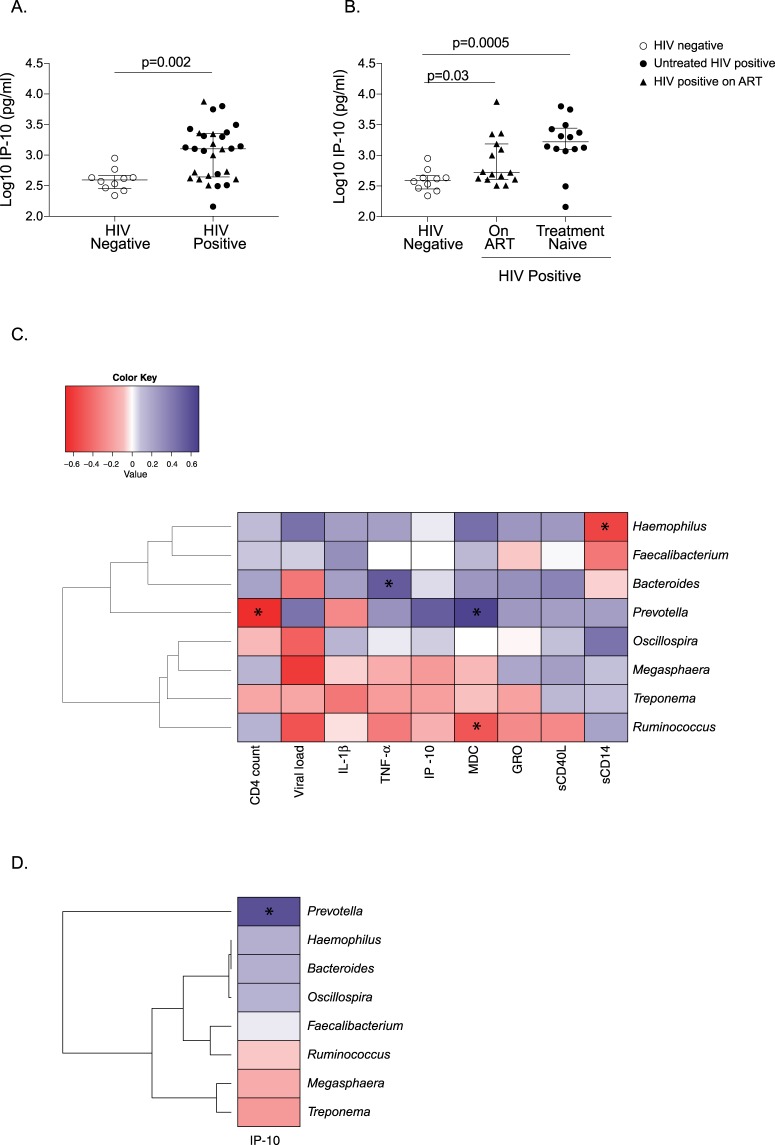
Comparison of Plasma IP-10 levels in (**A**) HIV positive and HIV negative children and (**B**) HIV negative, on ART and treatment naïve perinatally HIV-infected children. The horizontal line and deviation in the dot plot represents median and interquartile range (IQR), respectively. Mann-Whitney *U-*test and Kruskal-Wallis one-way ANOVA with Dunn’s multiple comparison were performed for statistical analysis. p < 0.05 was considered to be significant. (**C**) Heatmap representing the associations of relative abundance of significantly distinct genera with CD4 count, viral load, IL-1β, TNF-α, IP-10, GRO, MCD, sCDl40L and microbial translocation marker, sCD14. Blue and red shading represents positive and negative association respectively. Clustering was carried out based on the association of genera with clinical and immune parameters. (**D**) Heatmap representing the associations of absolute abundance of significantly distinct genera with IP-10. Blue shading indicates positive association and red shading indicates negative association. Clustering was carried out based on the association of genera with IP-10. Statistical analysis was carried out using Spearman *t*-test. p < 0.05 was considered to be significant. *p < 0.05.

**Figure 7:**
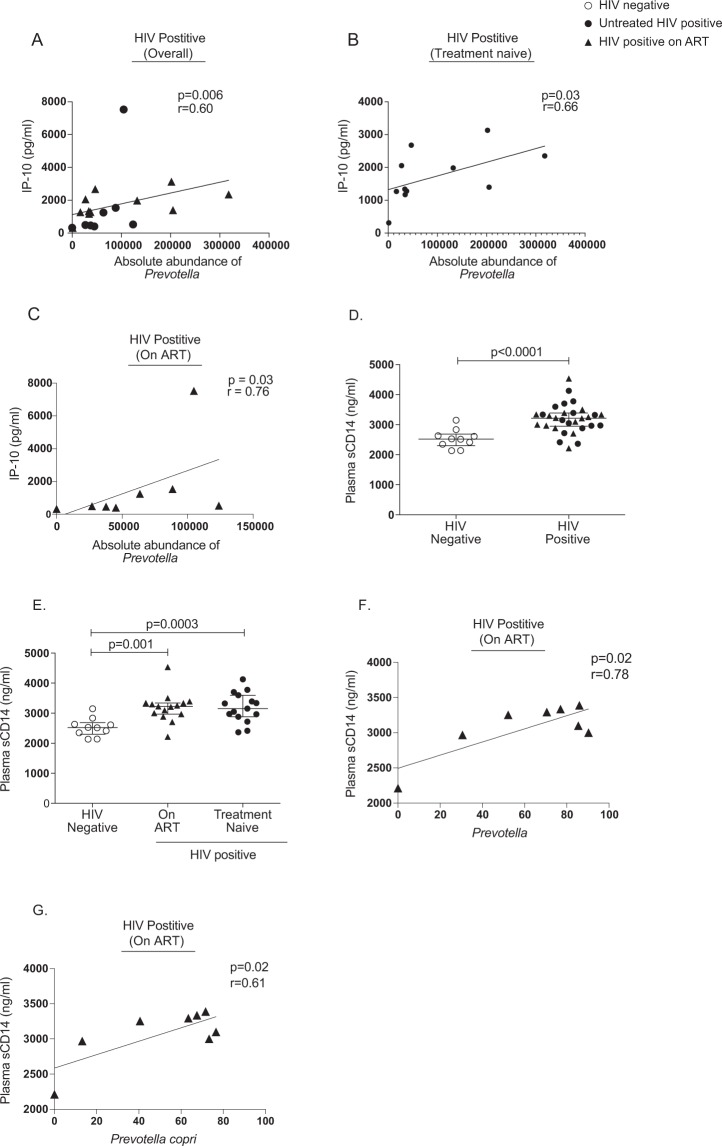
Association between absolute abundance of *Prevotella* and plasma IP-10 level (pg/ml) in (**A**) HIV positive children, (**B**) on ART and (**C**) treatment naïve perinatally HIV infected children. Statistical analysis was performed using Spearman *t*-test. A linear regression line is included in each plot. p < 0.05 was considered to be significant. Comparison of Plasma sCD14 levels in (**D**) HIV positive and HIV negative children and (**E**) HIV negative, on ART and treatment naïve perinatally HIV-infected children. The horizontal line and deviation in the dot plot represents median and interquartile range (IQR), respectively. Mann-Whitney *U-*test and Kruskal-Wallis one-way ANOVA with Dunn’s multiple comparison were performed for statistical analysis. p < 0.05 was considered to be significant. Association between (**F**) relative abundance of *Prevotella* and plasma sCD14 level (ng/ml) (**G**) relative abundance of *Prevotella copri* and plasma sCD14 level (ng/ml) in ART-suppressed perinatally HIV infected children. Statistical analysis was performed using Spearman *t*-test. A linear regression line is included in each plot. The p < 0.05 was considered to be significant.

Multiplex analysis revealed significantly high levels of IL-1β, TNF-α, IP-10 (Interferon-gamma inducible protein 10), MDC (Macrophage-derived chemokine), GRO (Growth regulated oncogene chemokine), and sCD40L (Soluble CD40-ligand) in treatment naïve HIV-infected children compared to uninfected controls. Treatment appeared to restore the levels of these cytokines except IP-10 (Table 1). The IP-10 level was significantly elevated in perinatally HIV-infected children (p = 0.002) and it remained elevated in treated group (p = 0.03) in comparison to uninfected controls (Fig. 6A,B). This suggests a potential role of IP-10 in maintaining the inflammatory environment in HIV-infected children on treatment. Next, we performed multivariate analysis to evaluate the association of significantly distinct genera with cytokines that were significantly elevated in HIV-infected children. Notably, *Prevotella* was directly correlated (r = 0.65, p = 0.005) and *Ruminococcus* was indirectly correlated (r = −0.49, p = 0.048) with MDC levels. *Bacteroides* showed direct association with TNF- α (r = 0.54, p = 0.02). Since, IP-10 was the only cytokine to be elevated despite therapy in HIV-infected children, we also evaluated the association of absolute abundance of significantly differentially abundant genera with IP-10 level. We found IP-10 levels directly correlated only with the absolute abundance of *Prevotella* in perinatally HIV-infected children with or without treatment (Figs 6D and 7A–C). The levels of IL-2, IL-3, IL-4, IL-5, IL-8, IL-9, IL-1RA, IL-13, IL-12p40, TNF-β, MIP-1β, Flt-3L, and MCP-3 were undetectable.

**Table 1:** Cytokine levels in perinatally HIV-infected children and HIV negative controls.

Nature	Analyte (pg/ml)	HIV-infected children		HIV negative control
		Treatment naïve Median (IQR)	On ART Median (IQR)	Median (IQR)
Proinflammatory	IL-1α	214.7 (113.6–334.4)	156.2 (81.44–266.8)	215.6 (59.57–326.4)
	IL-1β	**10.12 (0.0–12.03)**	5.345 (0.7575–12.30)	17.60 (4.888–30.79)
	IL-6	19.27 (10.54–40.71)	21.31 (0.0–32.84)	46.78 (5.043–63.64)
	IL-7	58.33 (41.91–69.07)	44.48 (37.38–52.47)	58.75 (35.05–76.12)
	IL-12p70	12.48 (0.7100–22.64)	8.625 (4.180–12.48)	27.60 (2.390–51.71)
	IL-15	9.810 (0.0–15.90)	7.055 (2.295–14.35)	19.12 (2.860–33.05)
	IL-17A	7.870 (4.070–13.29)	6.425 (2.080–11.26)	15.98 (4.440–23.43)
	INF-γ	23.15 (14.44–31.37)	19.37 (9.908–34.73)	34.06 (18.03–50.19)
	TNF-α	**103.9 (91.28–128.9)**	57.9 (47.76–75.61)	67.26 (45.94–77.95)
	GM-CSF	87.53 (52.17–104.5)	**54.9 (31.34–90.03)**	124.2 (57.50–167.2)
Anti-inflammatory	IL-10	28.85 (8.430–44.44)	11.60 (0.0–23.26)	33.35 (0.2075–47.67)
Chemoattractants	IP-10	**2582 (1739–4812)**	**884.7 (580–1936)**	569 (435.5–825.4)
	MCP-1	634 (591–746.3)	**473.7 (382.1–543)**	650.6 (482.3–806)
	MDC	**770.6 (546.3–883.2)**	466.5 (394.9–522.8)	573.4 (469.2–636.3)
	MIP-1α	39.42 (21.87–50.21)	36.93 (0.0–50.56)	39.13 (8.218–56.40)
	EOTAXIN	10.48 (8.040–12.66)	7.860 (0–13.89)	10.59 (3.463–15.64)
	Fractalkine	186.5 (59.9–227.2)	149 (118.6–211)	270.9 (95.77–416.2)
Growth Factors	VEGF	206.7 (117.7–329)	138.5 (86.69–252.2)	315.5 (119.7–481.2)
	EGF	20.82 (4.340–28.56)	20.70 (14.33–22.61)	15.25 (8.505–25.95)
	FGF-2	31.12 (17.25–36.75)	**21.28 (16.16–30.61)**	40.34 (22.49–57.55)
	TGF-α	0.1500 (0.0–0.5400)	0.2050 (0.0–0.5850)	0.7850 (0.1150–1.533)
	GRO	**2563 (1271–3056)**	582.2 (491.3–788.2)	581.9 (491.3–772.1)
	G-CSF	21.33 (17.14–27.44)	28.81 (22.48–38.60)	22.39 (15.23–30.01)
	INFα2	221.3 (123.8–275.4)	190.5 (132.8–231.5)	312.2 (160.8–432.1)
	sCD40L	**7247 (5101–8489)**	2023 (1549–4095)	3904 (2762–4629)

Bold numbers denote values different from HIV NEG, p < 0.05.

### Increased levels of plasma sCD14 in perinatally HIV-infected children despite ART

We evaluated the level of microbial translocation marker, sCD14 in the plasma of perinatally HIV-infected children. Plasma level of sCD14 was significantly elevated in perinatally HIV-infected children compared to uninfected controls (Fig. 7D). ART could not normalize the level of sCD14 in the plasma of treated HIV-infected children (Fig. 7E). Given that gut microbiota maintains gut epithelial barrier, we then evaluated whether significantly differentially abundant genera in HIV-infected children was associated with plasma level of sCD14. We found *Haemophilus* to be negatively associated with sCD14 (r = −0.56, p = 0.01) (Fig. 6C). Relative abundance of *Prevotella* and also *Prevotella copri* directly correlated with plasma sCD14 level in treated HIV-infected children (Fig. 7F,G).

## Discussion

In this paper, we studied the fecal microbiota in treatment-naïve and ART-suppressed perinatally HIV-infected children, and compared them with that of uninfected controls. Our study showed a unique pattern of fecal microbiota with elevated relative abundance of the genus *Prevotella* and species *Prevotella copri* in HIV-infected children that persisted during suppressive ART. The genus *Prevotella* was directly associated with soluble IP-10 and inversely correlated with CD4 count despite ART. The genus *Prevotella* and species *Prevotella copri* positively correlated with sCD14, a marker of microbial translocation, in ART-suppressed perinatally HIV-infected children implying that *Prevotella* may potentially be involved in persistent inflammation in perinatally HIV-infected children during therapy.

Alteration in the overall gut microbial diversity during HIV infection in adults currently remains controversial. Some studies reported gut microbiota to be less diverse in untreated HIV-infected adults^28,29,40,41^ or the diversity to be decreased after ART^28,36^, while others found no alteration in the diversity of gut microbiota^26,27,31,33,34^. In our study, we found decreased α-diversity in the fecal microbiota of HIV-infected children despite therapy. This discrepancy among previous studies could be due to different ethnicity, age, different stage of HIV infection of enrolled HIV-infected subjects, selection of sample type, use of antibiotics by study participants or different technologies used to study gut microbiota composition. Perinatally HIV-infected children and uninfected controls participated in our study had preserved CD4 count and shared similar geographical region and dietary habits. As antibiotics alter gut microbiota composition profoundly^42^, we only included children who were not on antibiotics within last one month of sample collection. Similar to studies on HIV-infected adults, beta diversity indices such as PCA showed composition of fecal microbiota in most of the perinatally HIV-infected children to be different from uninfected controls. Moreover, most of the treated HIV-infected children were clustered with treatment naïve HIV-infected children suggesting similar pattern of gut microbiota in treated and untreated perinatally HIV-infected children. Thus, despite successful viral suppression, ART could not completely restore fecal microbiota in children with HIV.

Untreated HIV-infected children had increased relative abundance of Bacteroidetes and decreased Firmicutes whereas studies on HIV-infected adults reported decreased Firmicutes with no difference in Bacteroidetes abundance compared to controls^25,29,30,43^. We did not observe any difference in the relative abundance of phylum Proteobacteria in perinatally HIV-infected children compared to uninfected controls, which is in contrast to a recent study by Dinh *et al*. who showed significantly enriched relative abundance of Proteobacteria in the fecal samples of adults living with HIV^31^. The variations in the relative abundance of Proteobacteria in HIV-infected subjects have been observed across studies. Some previous studies that evaluated intestinal mucosal biopsies showed increased relative abundance of Proteobacteria in HIV-positive patients^25,28^, whereas other failed to observe any difference in the fecal and rectal sponge samples of HIV-positive patients compared to healthy controls^26,29^.

Our observations of increased relative abundance of Prevotellaceae and decreased relative abundance of Bacteroidaceae and Rikenellaceae in untreated HIV-infected children are in agreement with the previous findings in HIV-infected adults^25,27,36^. Similar to HIV-infected adults^25–28,32^, we also observed significantly increased relative abundance of *Prevotella* with decreased abundance of *Bacteroide*s in perinatally HIV-infected children. Increase in gut *Prevotella* has been reported in chronic inflammatory diseases such as Rheumatoid arthritis^44,45^ and metabolic syndrome^46^. Recent studies have shown that the high abundance of *Prevotella* in HIV-infected individuals is attributed to their sexual behavior rather than to the disease itself^32,47^. However, in our study prior sexual behavior does not have any relevance because the study is focussed on perinatally HIV-infected children who have either not reached or just reached an early stage of puberty. A study by Dillon *et al*. showed positive correlation of relative abundance of *Prevotella* with mucosal CD4+ and CD8+ T cell activation, and pro-inflammatory cytokine-producing mucosal CD4+ and CD8+ T cells^25^ however, Ling *et al*. did not find any significant correlation between *Prevotella* abundance and systemic inflammatory cytokines^27^ in HIV-infected adults. This suggests that the potential inflammatory role of *Prevotella* is only confined to the local gut immune system. In contrast to the previous study in adults^25,27^, we found that relative abundance of *Prevotella* was indirectly associated with peripheral CD4 count and directly associated with MDC in perinatally HIV-infected children. MDC has emerged as novel modulator of lung inflammation in mice^48^ and an inflammatory marker to assess the severity of atopic dermatitis in children and infants^49^. More recently, the increased levels of MDC in HIV-infected adults have also been reported^50^. This suggests the potential inflammatory role of MDC in HIV-infected children, however the level of MDC normalizes after initiation of ART. Interestingly, absolute abundance of *Prevotella* was found to be directly associated with soluble IP-10 in untreated and ART-experienced perinatally HIV-infected children. Our results indicate that *Prevotella* outgrowth (represented by increase in the absolute number of *Prevotella*), rather than *Prevotella* evenness may drive the production of IP-10.

Level of IP-10 has been shown to be elevated in many viral infections, including acute West Nile virus^51^, acute and chronic HCV^52,53^ and severe influenza infection^54,55^ suggesting the role of this chemokine in immune response to viral infection. Elevated IP-10 has been reported during acute and chronic HIV infection^52,56–59^. Consistent with previous studies on HIV-infected adults^60,61^, ART could not normalise the plasma level of IP-10 in perinatally HIV-infected children to that of uninfected controls. Previous studies on acute and chronic HIV infection in adults showed positive correlation of IP-10 with viral load^57,59^ and negatively with CD4 count^56^, thus proposed IP-10 to be the marker of disease progression. However, we failed to find direct correlation between IP-10 and HIV viral load in perinatally HIV-infected children, possibly because of limited sample size.

Infection of monocyte-derived macrophages (MDM) with HIV-1 has been shown to stimulate the production of GRO-α chemokine *in-vitro*. This chemokine in turn prompt HIV replication in macrophages and lymphocytes, thereby contributing to HIV pathogenesis^62^. Our observation of elevated level of GRO in untreated HIV-infected children might be due to viral replication.

Elevated sCD14, a marker of microbial translocation is associated with increased mortality among HIV-infected individuals^63^ and also sCD14 level in HIV-infected mothers have reported to be directly associated with risk of mother to child transmission^64^. Consistent with prior findings^65–68^, perinatally HIV-infected children had increased plasma level of sCD14 compared to uninfected children, showing gut epithelial barrier disruption and microbial translocation that persists even after suppressive therapy. Our study also showed positive correlation of sCD14 with abundance of genus *Prevotella* and *Prevotella copri* in treated HIV-infected children. However the presence of virus appears to disrupt this correlation as seen in untreated HIV-infected children. This suggests that either HIV or other unknown factors driven by HIV viral load contribute to microbial translocation in untreated subjects. Additional contributory factors to increased microbial translocation in untreated children are the decreased relative abundance of Lachnospiracea and Ruminococcacea families that include many butyrate-producing bacterial species that are essential for preserving the gut mucosal integrity^69^.

Ours is the first study to investigate the fecal microbiota composition of treatment naïve and ART-suppressed perinatally HIV-infected children that showed association of genus *Prevotella* with IP-10 and sCD14, a microbial translocation marker. These results suggest that *Prevotella* is proinflammatory in nature that may augment the inflammation in perinatally HIV–infected children despite ART, and may potentially promote chronic complications. Our study has few limitations. First, in our cross sectional study fecal microbiota has been used as a proxy for gut microbiota. Fecal microbiota only represents gut lumen microbiota and does not reflect mucosal-associated microbiota, which is important to consider as microbes on the gut mucosa can potentially interact with gut associated lymphoid tissue (GALT). However fecal sample was only the realistic sample for non-invasive study. Second, though we showed impact of ART on the fecal microbiota of HIV-infected children, the effect of individual antiretroviral drugs on fecal microbiota is not investigated in our study.

In summary, our study found distinct gut microbiota composition in untreated and ART-suppressed perinatally HIV-infected children compared to uninfected controls. They had higher abundance of *Prevotella* and decreased *Bacteroides*, *and* elevated levels of IP-10 that persists despite ART. The higher abundance of *Prevotella* may promote HIV pathogenesis as suggested by the negative association of *Prevotella* with CD4 count. Interestingly, the direct correlation between *Prevotella* and IP-10 suggests that IP-10 may be regulated by gut microbiota and could potentially contribute to ongoing inflammation and T cell depletion. Future studies to understand the mechanism of *Prevotella*-driven inflammation may aid the development of therapeutic interventions that can reconstitute healthy gut microbiota and mitigate the level of inflammation in individuals living with HIV.

## Methods

### Study Population

Study population included 29 perinatally HIV-1-infected children and 14 uninfected age matched controls. Perinatal infection in the child was confirmed by the documentation of HIV infection within 1 year of life. Among HIV-infected children, 15 children were on ART for more than 6 months, whereas the other 14 children were not on ART (treatment naïve). To study the gut microbiota, 11-treatment naïve and 8 ART-suppressed perinatally HIV-infected children were included together with 14 uninfected controls for the high throughput sequencing of DNA extracted from fecal sample. All treated HIV-infected children had undetectable plasma viral load (VL < 50 copies/ml). Uninfected controls were matched for sex and age to HIV-infected children. The following criteria were used to exclude children: age more than 15 years old, use of antibiotics or probiotics within one month of sample collection, any medical history of intestinal inflammatory disorders, evidence of hepatitis B and C infection, and vaccination within 1 week of sample collection. All study participants belonged to same ethnicity, geographical area and had similar dietary habits. The study was approved by Institutional Review Board (IRB) of St. Johns Medical College & Hospital at Bangalore, Jawaharlal Nehru University (JNU) at New Delhi and King George’s Medical University (KGMU) Lucknow, and was carried out in accordance with the approved guidelines. Informed written consent was obtained from parents/guardian of all the children who participated in our study. The characteristics of subjects are listed in the Table 2.

**Table 2:** Characteristics of subjects.

Parameters	HIV-infected children		HIV negative control
	Treatment naïve	ART-suppressed	
Number	14	15	14
Age (years)	10 (8–12)	11 (7–12)	10 (7–15)
Male % (n)	78.5 (11)	40 (6)	60 (7)
Female % (n)	21.5 (3)	60 (9)	40 (7)
Male/Female ratio	3.6	0.6	1
CD4 count (cells per µl)	644 (330–1334)	1141.5 (523–2406)	1343 (863–1884)
Plasma viral load (HIV-1 RNA copies/ml)	33,779 (1,461–4,31,237)	<50	NA

Values are shown as median (IQR), NA: Not applicable.

### Sample collection

Approximately 2–3 g of fresh fecal sample was collected in a sterile plastic container from each child and stored at −80 °C within 30 minutes. Peripheral blood was collected into vacutainer blood collection tubes containing EDTA or Heparin and whole blood was centrifuged to isolate plasma. Plasma was stored at −80 °C for measuring levels of cytokines.

### Plasma viral load and CD4 count

Plasma HIV-1 viral load (VL) was measured using Abbott Real Time HIV-1 assay with a lower limit of detection of 50 copies of RNA/ml (Abbott Molecular Inc., Des Plaines, IL, USA. CD4 T cell count was measured using FC500™ flow cytometer (Beckman Coulter, Fullerton, California, USA).

### Fecal DNA extraction

Fecal sample stored at −80 °C was thawed on ice and approximately 220 mg of fecal sample was taken in a microcentrifuge tube for bacterial DNA isolation. Microbial DNA was isolated from fecal samples by commercially available QIAamp Fast DNA Stool Mini kit, as per manufacturer’s recommended protocol. The amount and purity of DNA were estimated by absorbance at 260 nm and 280 nm using nanodrop spectrophotometer.

### Amplicon Library preparations and Sequencing

Identification of bacteria was carried out by amplifying and sequencing bacterial 16S rRNA gene at Eurofins Genomics India Pvt Ltd (Bangalore, India). Oligonucleotide primers (Forward - 5′ GCCTACGGGNGGCWGCAG 3′ and Reverse-5′ ACTACHVGGGTATCTAATCC 3′) that target V3-V4 region of 16S rRNA gene were used to generate amplicons. The amplicon libraries were prepared using Nextera XT Index Kit (Illumina inc.) as per the 16S Metagenomic Sequencing Library preparation protocol and also include multiplexing index sequences and illumina adapters. The amplicon libraries were purified by 1X AMpureXP beads, quantified using Qubit fluorometer and analyzed in 4200 Tape Station system (Agilent Technologies) using D1000 Screen tape as per manufacturer instructions. Sequencing of the amplicons was performed on Miseq Platform using 2 × 300 v3 Miseq Kit.

### Luminex cytokine assays

A Luminex cytokine/chemokine assay (EMD Millipore) was used to examine the plasma samples of 14-treatment naïve, 15 ART-suppressed HIV-infected children and 10 uninfected controls. The assay measured the following serum cytokine/chemokine levels: IL-1α, IL-1β, IL-2, IL-3, IL-4, IL-5, IL-6, IL-7, IL-8, IL-9, IL-10, IL-12p40, IL-12p70, IL-13, IL-15, IL-17A, INF-γ, TNF-α, TNF-β, GM-CSF, IL-1RA, IP-10, MCP-1, MDC, MIP-1β, MIP-1β, EOTAXIN, Fractalkine, VEGF, EGF, FGF-2, TGF-β, GRO, G-CSF, Flt-3L, INF-α2, MCP-3 and sCD40L. Cytokine standards and each sample were tested in duplicate. Data was acquired on Bio-Plex 200 system using Bio-Plex manager software, v4.1 (Bio-Rad).

### sCD14 detection

Level of sCD14 in plasma was quantified by commercially available Human sCD14 Elisa kit according to the manufacturers’ protocols (R&D Systems, Minneapolis, MN). Each sample was analysed in duplicate and sCD14 level was expressed in ng/ml.

### Sequence data and Statistical analysis

Sequencing data was analysed using QIIME 1.9.1^70^. Raw reads were quality filtered using Trimmomatic v0.38. Quality control involved the removal of reads with errors in barcode or primer sequences, reads containing ambiguous bases and low quality sequences (reads with more than 10% quality threshold (QV) <20 phred score) along with a sliding window of 20 bp and a minimum length of 100 bp. According to barcodes, the high quality reads obtained were then assigned to samples. Using UCLUST in QIIME the high quality sequences were clustered into operational taxonomic units (OTUs) with 97% sequence similarity. OTUs was used to analyze alpha diversity indices (Shannon and Simpson) using QIIME. For PCA, distance matrices were calculated using R. Using Greengenes reference databases, the taxonomies are assigned to the OTUs by UCLUST with a threshold of 90% sequence similarity. Explicet software v2.8.5 was used to analyze OTUs and create figures^71^. Significant differences in the overall relative abundance of fecal microbiota at phylum, family and genus level between treatment naïve, ART-suppressed HIV-infected children and uninfected controls were estimated using adonis function of vegan R package^72^. Statistical analysis of alpha diversity indices, the relative abundance of taxa, plasma cytokines, and soluble sCD14 levels between two and three groups was performed using non-parametric Mann Whitney U test and Kruskal-Wallis 1-way ANOVA with Dunn’s multiple comparison respectively in GraphPad Prism v. 6. LEfSe analysis (http://huttenhower.sph.harvard.edu/lefse/) was used to identify differentially abundant bacterial taxa across three groups with alpha value of 0.05 and LDA score of >2.4^73^. Association between two variables was carried out by Spearman rank correlation test. Correlation coefficient r = 1, describes a positive association and r = −1, describes a negative correlation. A p value below 0.05 was considered significant. Heatmap was generated to visualize association between two variables using heatmap.2 function of gplots R package (https://CRAN.R-project.org/package=gplots).

## Electronic supplementary material

Supplementary information
