# Supplementary material for: High Abundance of genus Prevotella in the gut of perinatally HIV-infected children is associated with IP-10 levels despite therapy
Source: Sci Rep. 2018 Dec 5;8:17679. doi: 10.1038/s41598-018-35877-4 (PMC6281660; doi:10.1038/s41598-018-35877-4)
Supplement: Supplementary file 1 — Supplementary information [file 41598_2018_35877_MOESM1_ESM.pdf]

High Abundance of genus *Prevotella* in the gut of perinatally HIV-infected children is associated with IP-10 levels despite therapy

Urvinder Kaur S[1], Anita Shet[2], Niharika Rajnala[3], Bindu Parachalil Gopalan[3], Preeti Moar[1], Himanshu D[4], Balendra Pratap Singh[5], Rupesh Chaturvedi[6], Ravi Tandon[1#]

[1]Laboratory of AIDS Research and Immunology, School of Biotechnology, Jawaharlal Nehru University, New Delhi, India; [2]International Vaccine Access Center, Johns Hopkins School of Public Health, Baltimore, USA; [3]Division of Infectious Diseases, St. John's Research Institute, St. John's National Academy of health Sciences, Bangalore, India; [4]Department of Medicine, King Georges Medical University, Lucknow, India; [5]Department of Prosthodontics, King Georges Medical University, Lucknow, India; [6]Host Pathogen Interaction Laboratory, School of Biotechnology, Jawaharlal Nehru University, New Delhi, India

# Corresponding Author:

Ravi Tandon
School of Biotechnology
Jawaharlal Nehru University
New Delhi-110067, India
Phone: +91-11-26738894
Fax: +91-11-26717586
E-mail: ravitandon@jnu.ac.in

Figure S1

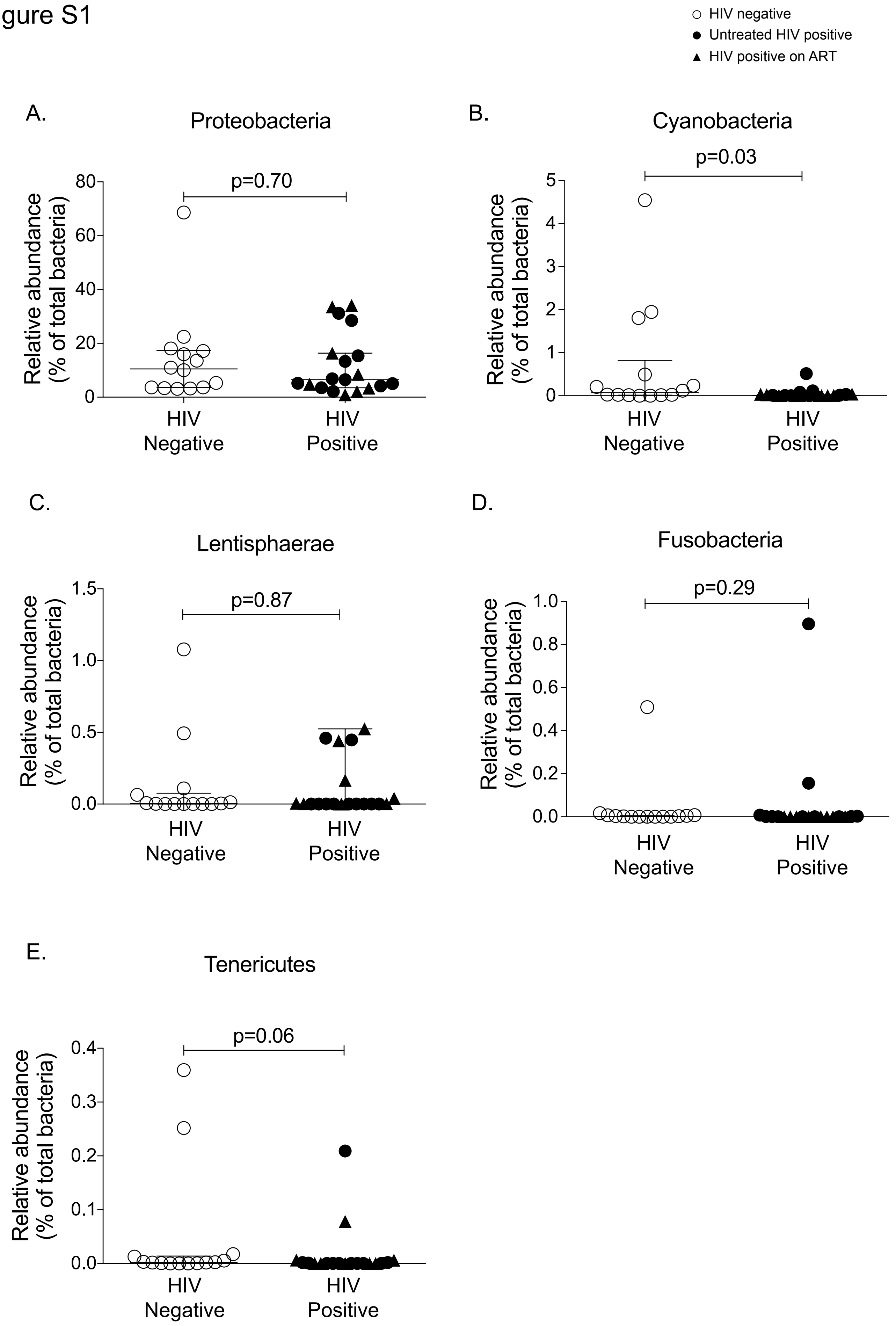

○ HIV negative
● Untreated HIV positive
▲ HIV positive on ART
A.
Proteobacteria
p=0.70
Relative abundance (% of total bacteria)
0
20
40
60
80
HIV Negative
HIV Positive
B.
Cyanobacteria
p=0.03
Relative abundance (% of total bacteria)
0
1
2
3
4
5
HIV Negative
HIV Positive
C.
Lentisphaerae
p=0.87
Relative abundance (% of total bacteria)
0.0
0.5
1.0
1.5
HIV Negative
HIV Positive
D.
Fusobacteria
p=0.29
Relative abundance (% of total bacteria)
0.0
0.2
0.4
0.6
0.8
1.0
HIV Negative
HIV Positive
E.
Tenericutes
p=0.06
Relative abundance (% of total bacteria)
0.0
0.1
0.2
0.3
0.4
HIV Negative
HIV Positive

Figure S2

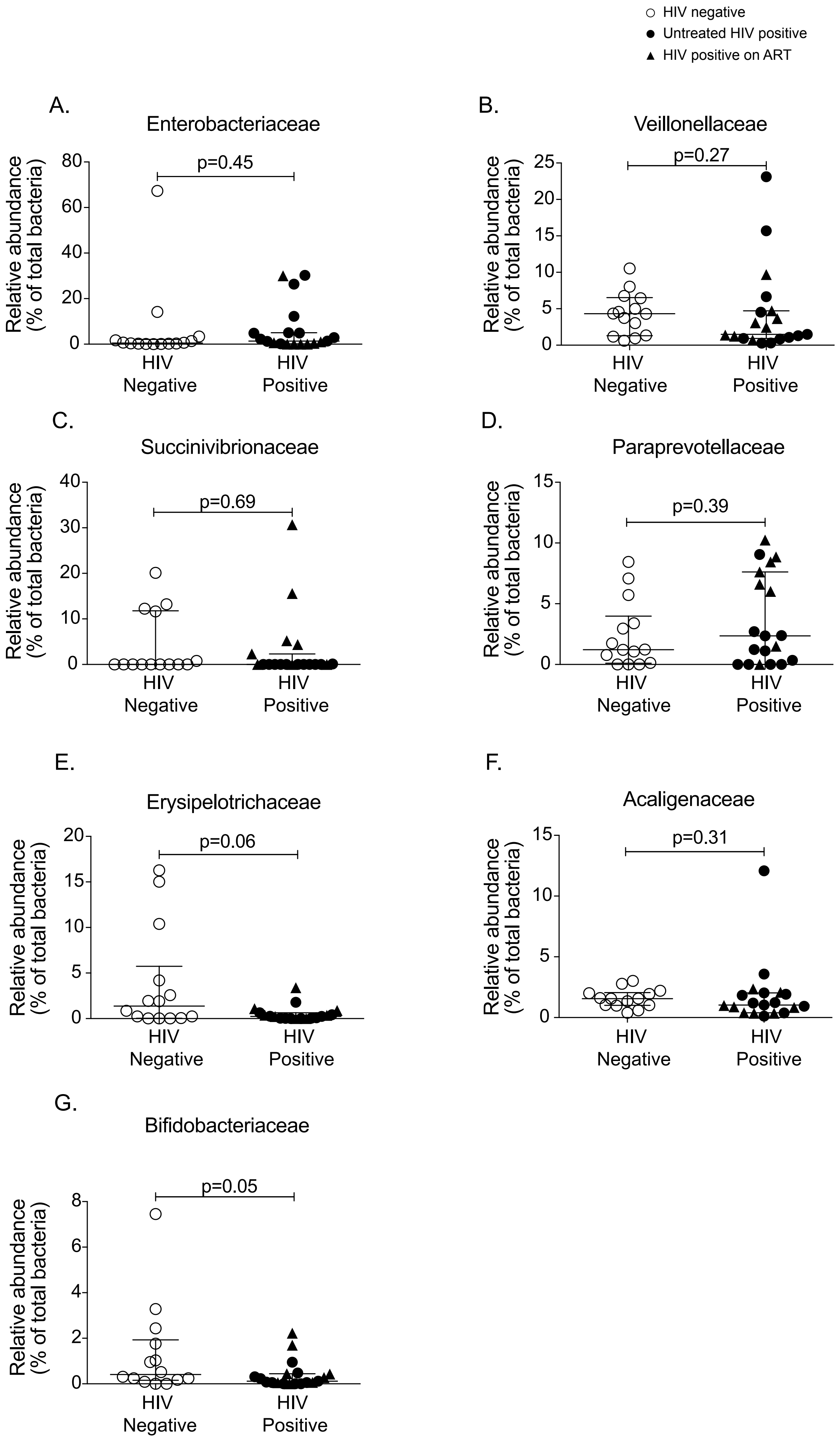

HIV negative
Untreated HIV positive
HIV positive on ART
A.
Enterobacteriaceae
p=0.45
Relative abundance (% of total bacteria)
80
60
40
20
0
HIV Negative
HIV Positive
B.
Veillonellaceae
p=0.27
25
20
15
10
5
0
C.
Succinivibrionaceae
p=0.69
40
30
20
10
0
D.
Paraprevotellaceae
p=0.39
15
10
5
0
E.
Erysipelotrichaceae
p=0.06
20
15
10
5
0
F.
Acaligenaceae
p=0.31
15
10
5
0
G.
Bifidobacteriaceae
p=0.05
8
6
4
2
0

Figure S3

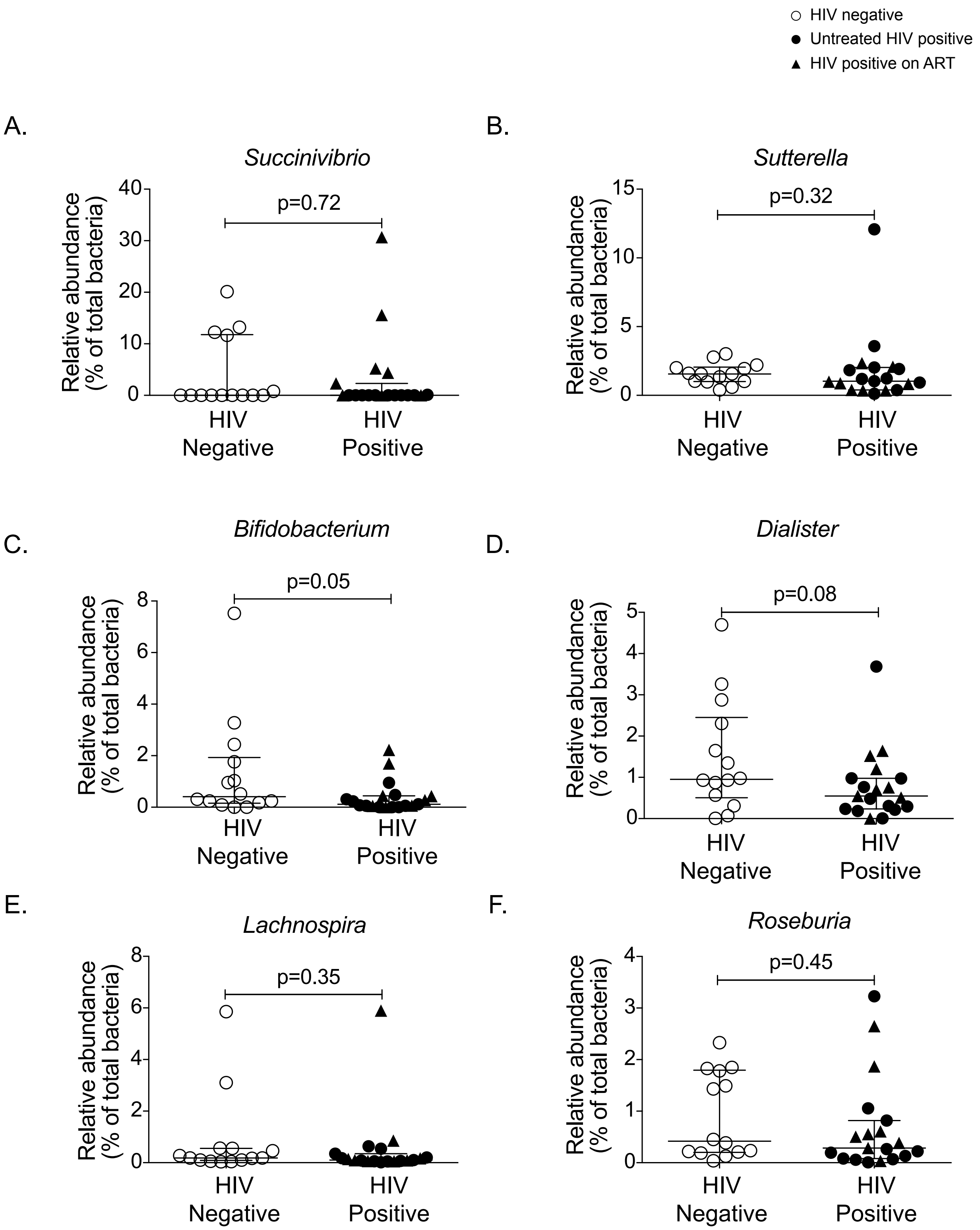

HIV negative
Untreated HIV positive
HIV positive on ART
A.
Succinivibrio
p=0.72
Relative abundance (% of total bacteria)
40
30
20
10
0
HIV Negative
HIV Positive
B.
Sutterella
p=0.32
15
10
5
0
HIV Negative
HIV Positive
C.
Bifidobacterium
p=0.05
8
6
4
2
0
HIV Negative
HIV Positive
D.
Dialister
p=0.08
5
4
3
2
1
0
HIV Negative
HIV Positive
E.
Lachnospira
p=0.35
8
6
4
2
0
HIV Negative
HIV Positive
F.
Roseburia
p=0.45
4
3
2
1
0
HIV Negative
HIV Positive

Figure S4

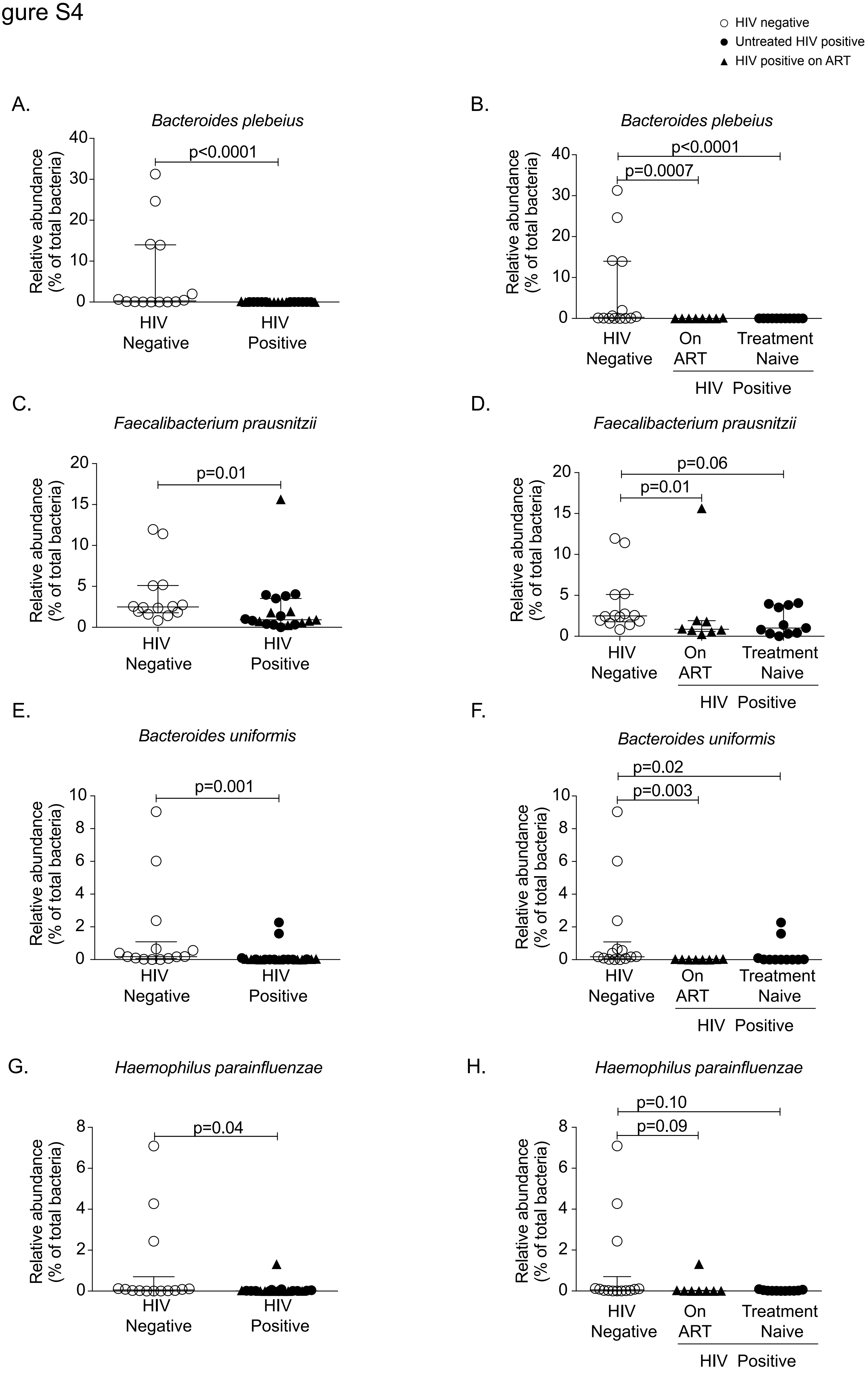

HIV negative
Untreated HIV positive
HIV positive on ART
A.
Bacteroides plebeius
Relative abundance (% of total bacteria)
p<0.0001
HIV Negative
HIV Positive
B.
Bacteroides plebeius
p<0.0001
p=0.0007
HIV Negative
On ART
Treatment Naive
HIV Positive
C.
Faecalibacterium prausnitzii
p=0.01
D.
Faecalibacterium prausnitzii
p=0.06
p=0.01
E.
Bacteroides uniformis
p=0.001
F.
Bacteroides uniformis
p=0.02
p=0.003
G.
Haemophilus parainfluenzae
p=0.04
H.
Haemophilus parainfluenzae
p=0.10
p=0.09

Figure S5

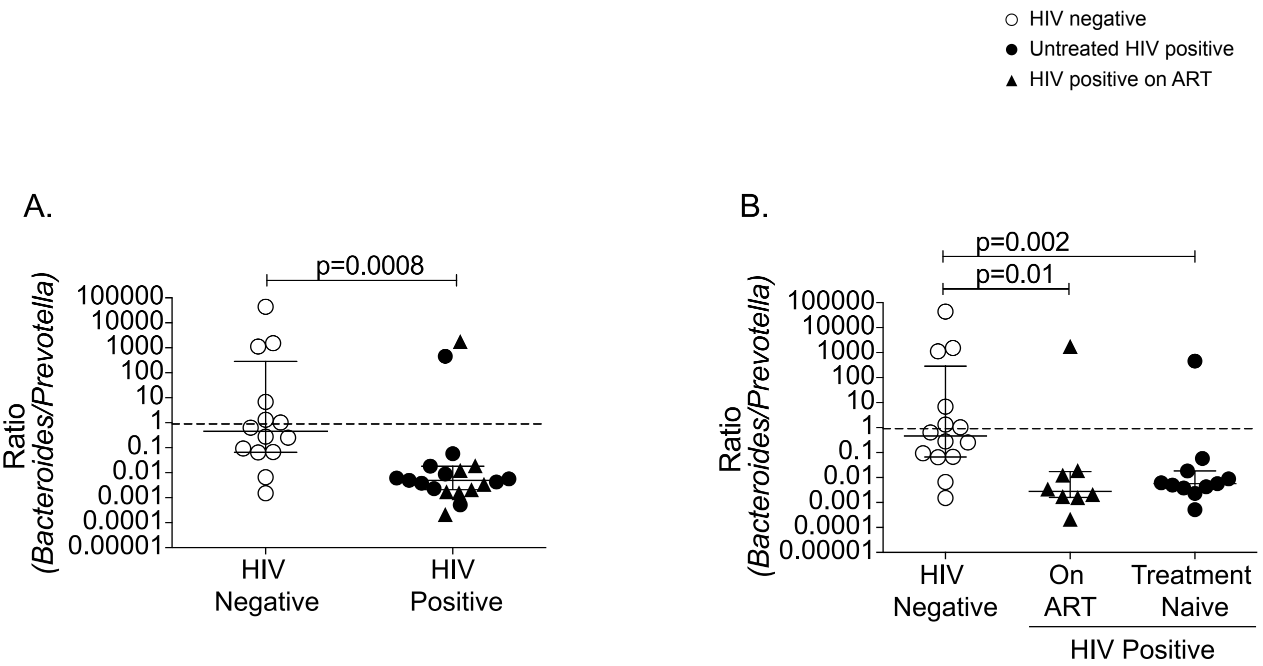

HIV negative
Untreated HIV positive
HIV positive on ART
A.
p=0.0008
Ratio (Bacteroides/Prevotella)
100000
10000
1000
100
10
1
0.1
0.01
0.001
0.0001
0.00001
HIV Negative
HIV Positive
B.
p=0.002
p=0.01
Ratio (Bacteroides/Prevotella)
100000
10000
1000
100
10
1
0.1
0.01
0.001
0.0001
0.00001
HIV Negative
On ART
Treatment Naive
HIV Positive

Table S1

	HIV Positive	HIV Negative	
	**Median (IQR)**	**Median (IQR)**	**p value**
**Phylum**			
Bacteroidetes	80.03 (56.53 – 87.19)	52.05 (40.66 – 57.70)	0.003
Firmicutes	9.406 (7.388 – 18.51)	36.36 (23.40 – 40.57)	0.002
Actinobacteria	0.195 (0.097 – 0.487)	1.165 (0.409 – 2.888)	0.0007
Spirochaetes	0.001 (0.0 – 0.002)	0.0 (0.0 – 0.0)	0.0007
Cyanobacteria	0.009 (0.004 – 0.037)	0.074 (0.016 – 0.821)	0.03
**Family**			
Prevotellaceae	70.35 (44.84 – 82.96)	28.15 (1.235 – 39.7)	0.0007
Bacteroidaceae	0.33 (0.166 – 0.56)	9.456 (2.151 – 26.02)	0.0008
Ruminococcaceae	3.034 (1.862 – 5.9)	11.31 (4.814 – 19.02	0.005
Lachnospiraceae	1.374 (0.721 – 3.238)	4.429 (1.516 – 7.3)	0.01
Rikenellaceae	0.003 (0.001 – 0.02)	0.33 (0.145 – 1.452)	<0.0001
**Genus**			
Prevotella	70.35 (44.84 – 82.96)	28.15 (1.235 – 39.7)	0.0007
Bacteroides	0.33 (0.166 – 0.56)	9.456 (2.151 – 26.02)	0.0008
Faecalibacterium	0.9185 (0.409 – 3.535)	2.492 (1.776 – 5.116)	0.01
Megasphaera	0.57 (0.002 – 2.784)	0.001 (0.0 – 0.486)	0.02
Ruminococcus	0.136 (0.022 – 0.64)	0.63 (0.244 – 6.088)	0.006
Haemophilus	0.008 (0.003 – 0.03)	0.06 (0.012 – 0.736)	0.04
**Species**			
Prevotella copri	67.45 (40.52 – 76.72)	17.69 (1.121 – 37.44)	0.001

Table S2

	Treatment naive	On ART	HIV Negative
	Median (IQR)	Median (IQR)	Median (IQR)
**Phylum**			
Bacteroidetes	*84.25 (58.48 – 88.83)	74.42 (40.08 – 86.93)	52.05 (40.66 – 57.70)
Firmicutes	*8.56 (5.816 – 12.85)	11.08 (8.593 – 40.91)	36.36 (23.4 – 40.57)
Actinobacteria	*0.134 (0.099 – 0.357)	0.395 (0.075 – 1.528)	1.165 (0.409 – 2.888)
Spirochaetes	*0.0002 (0.0 – 0.002)	* (0.0 – 0.002)	0.0 (0.0 – 0.0)
**Family**			
Prevotellaceae	*81.6 (54.41 – 86.15)	*69.69 (27.9 – 78.88)	28.15 (1.23 – 39.7)
Bacteroidaceae	*0.409 (0.234 – 1.08)	*0.216 (0.122 – 0.526)	9.456 (2.15 – 26.02)
Ruminococcaceae	*4.747 (1.14 – 5.545)	2.643 (1.989 – 18.07)	11.31 (4.814 – 19.02)
Lachnospiraceae	*1.179 (0.524 – 1.678)	1.976 (0.944 – 7.063)	4.429 (1.516 – 7.301)
Rikenellaceae	*0.004 (0.002 – 0.012)	*0.002 (0.0 – 0.03)	0.33 (0.144 – 1.452)
**Genus**			
Prevotella	*81.6 (54.41 – 86.15)	*69.69 (27.9 – 78.88)	28.15 (1.23 – 39.7)
Bacteroides	*0.409 (0.234 – 1.08)	*0.216 (0.122 – 0.526)	9.456 (2.15 – 26.02)
Faecalibacterium	1.001 (0.318 – 3.829)	*0.857 (0.592 – 1.906)	2.492 (1.776 – 5.116)
Megasphaera	*0.573 (0.004 – 5.106)	0.548 (0.0009 – 2.682)	0.001 (0.0 – 0.486)
Ruminococcus	*0.047 (0.014 – 0.136)	0.343 (0.152 – 2.157)	0.633 (0.245 – 3.05)
Haemophilus	0.012 (0.006 – 0.044)	0.008 (0.0 – 0.03)	0.063 (0.012 – 0.737)
**Species**			
Prevotella copri	*68.21 (45.77 – 82.94)	*65.47 (20.01 – 72.85)	17.69 (1.121 – 37.44)

*values that are significantly different from HIV negative controls ($p<0.05$)

Supplementary figure and table legends:

Table S1: Relative abundance of significantly distinct fecal microbiota between perinatally HIV-infected children and HIV negative controls at phylum, family, genus and species levels.

Table S2: Relative abundance of significantly distinct fecal microbiota across treatment naïve, On ART perinatally HIV-infected children and HIV negative controls at phylum, family, genus and species levels.

Figure S1: Plots representing relative abundance of **A.** Proteobacteria **B.** Cyanobacteria **C**. Lentisphaerae **D**. Fusobacteria and **E**. Tenericutes phyla in HIV negative and HIV positive children. The horizontal line and deviation in the dot plot represents median and interquartile range (IQR), respectively. Mann-Whitney-U-test was performed for statistical analysis. $p<0.05$ was considered to be significant.

Figure S2: Plots representing relative abundance of **A.** Enterobacteriaceae **B.** Veillonellaceae **C**. Succinivibrionaceae **D**. Paraprevotellaceae **E**. Erysipelotrichaceae **F**. Acaligenaceae and **G**. Bifidobacteriaceae families in HIV negative and HIV positive children. The horizontal line and deviation in the dot plot represents median and interquartile range (IQR), respectively. Mann-Whitney-U-test was performed for statistical analysis. $p<0.05$ was considered to be significant.

Figure S3: Plots representing relative abundance of **A.** *Succinivibrio* **B.** *Sutterella* **C**. *Bifidobacterium* **D**. *Dialister* **E**. *Lachnospira* and **F**. *Roseburia* genera in HIV negative and HIV positive children. The horizontal line and deviation in the dot plot represents median and interquartile range (IQR), respectively. Mann-Whitney-U-test was performed for statistical analysis. $p<0.05$ was considered to be significant.

Figure S4: Plots representing relative abundance of **A.** *Bacteroides plebeius* **C.** *Faecalibacterium prausnitzii* **E**. *Bacteroides uniformis* and **G**. *Haemophilus parainfluenzae* species in HIV negative and HIV positive children. Plots representing relative abundance of **B.** *Bacteroides plebeius* **D.** *Faecalibacterium prausnitzii* **F**. *Bacteroides uniformis* and **H**. *Haemophilus parainfluenzae* species across HIV negative controls, On ART and treatment naive HIV positive children. The horizontal line and deviation in the dot plot represents median and interquartile range (IQR), respectively. Mann-Whitney *U*-test and Kruskal-Wallis one-way ANOVA with Dunn's multiple comparison were performed for statistical analysis. $p<0.05$ was considered to be significant.

Figure S5: Plot showing the ratio of the relative abundance of *Bacteroides* to *Prevotella* A. between HIV negative and HIV positive children **B**. across HIV negative controls, On ART and treatment naive HIV positive children. Dotted line shows a ratio of 1.0 (relative abundance of *Bacteroides* = relative abundance of *Prevotella*). The horizontal line and deviation in the dot plot represents median and interquartile range (IQR), respectively. Mann-Whitney *U*-test and Kruskal-Wallis one-way ANOVA with Dunn's multiple comparison were performed for statistical analysis

Figure S1

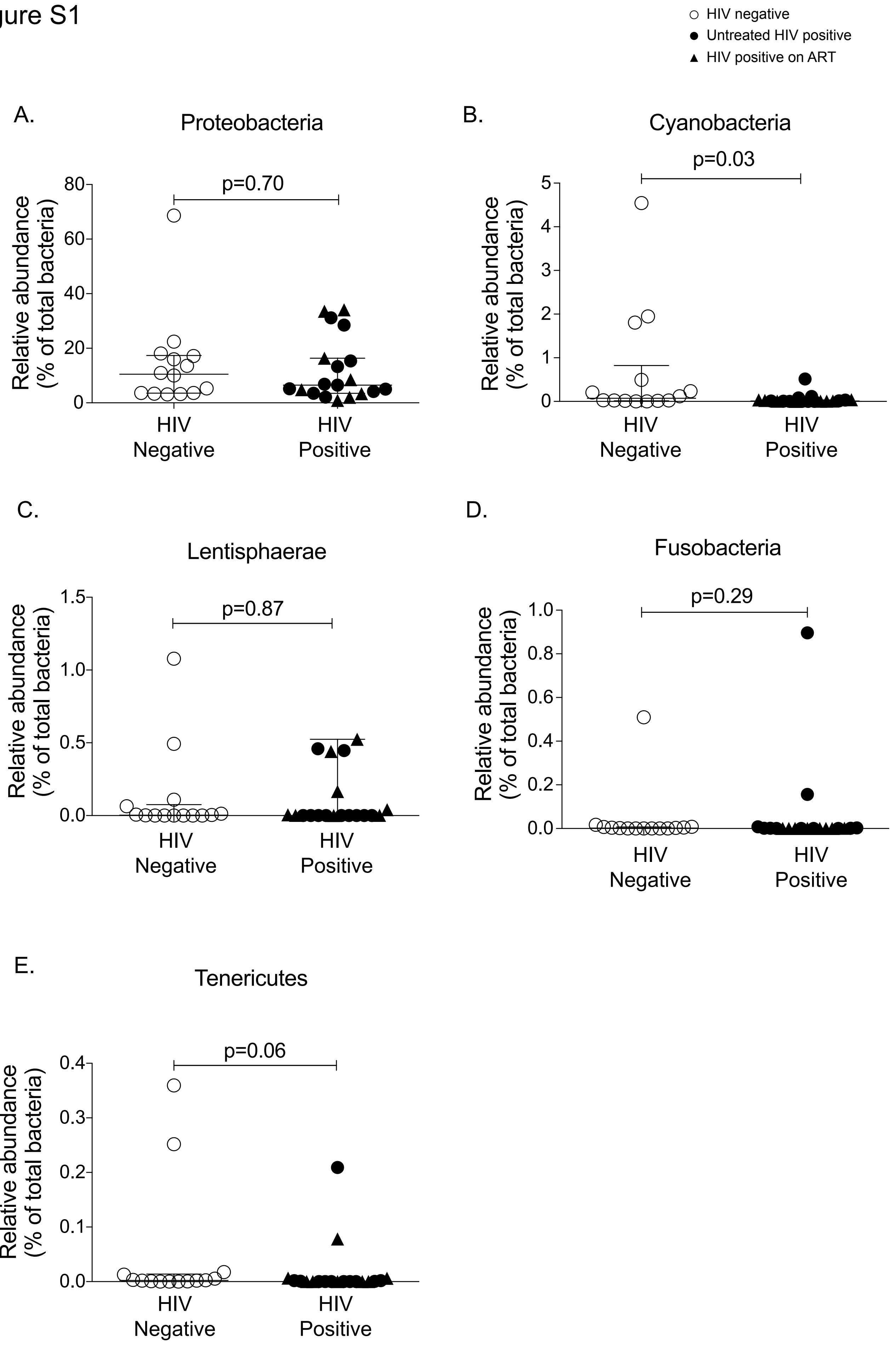

HIV negative
Untreated HIV positive
HIV positive on ART
A.
Proteobacteria
p=0.70
Relative abundance
(% of total bacteria)
80
60
40
20
0
HIV
Negative
HIV
Positive
B.
Cyanobacteria
p=0.03
Relative abundance
(% of total bacteria)
5
4
3
2
1
0
HIV
Negative
HIV
Positive
C.
Lentisphaerae
p=0.87
Relative abundance
(% of total bacteria)
1.5
1.0
0.5
0.0
HIV
Negative
HIV
Positive
D.
Fusobacteria
p=0.29
Relative abundance
(% of total bacteria)
1.0
0.8
0.6
0.4
0.2
0.0
HIV
Negative
HIV
Positive
E.
Tenericutes
p=0.06
Relative abundance
(% of total bacteria)
0.4
0.3
0.2
0.1
0.0
HIV
Negative
HIV
Positive

Figure S2

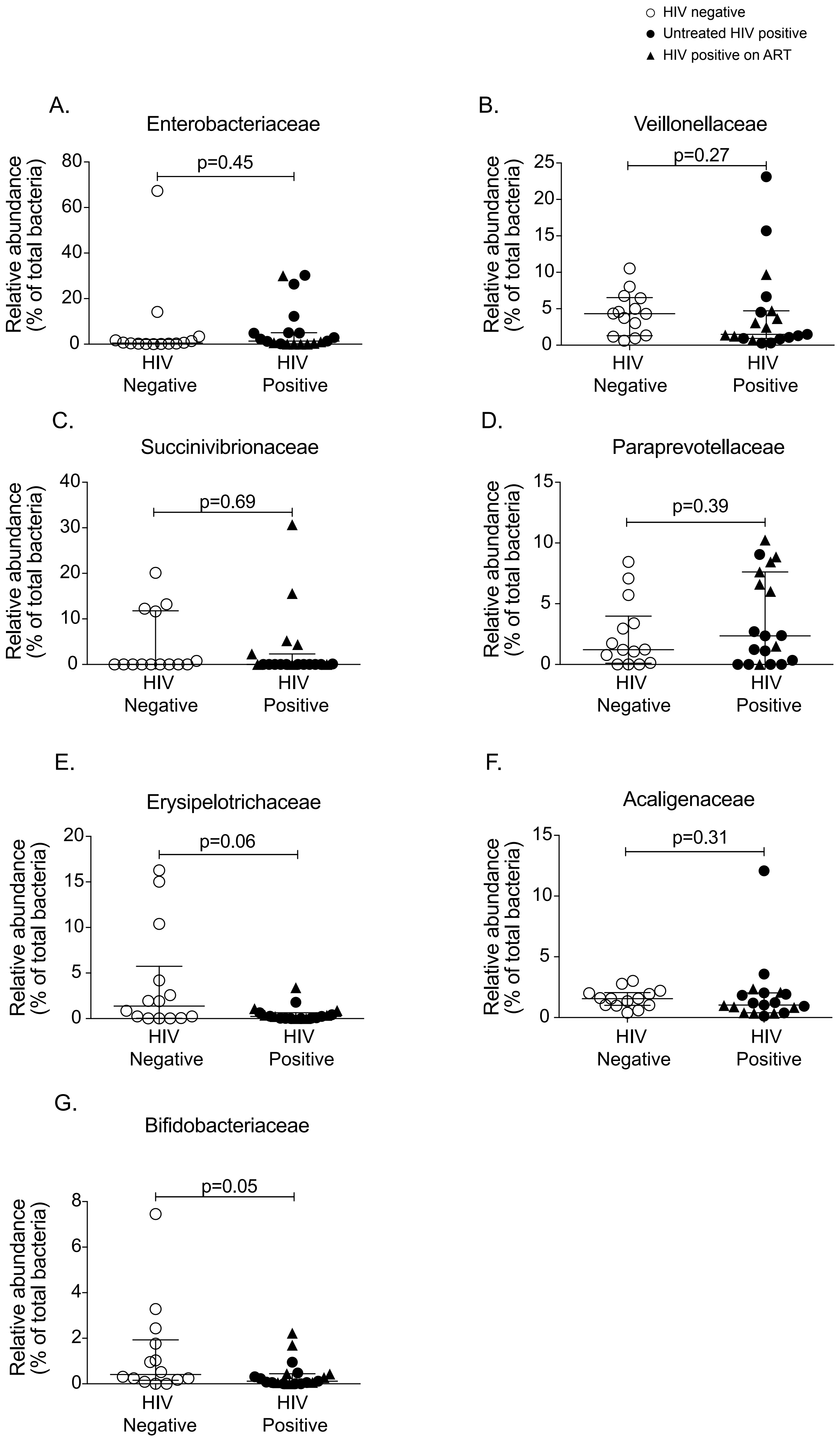
○ HIV negative
● Untreated HIV positive
▲ HIV positive on ART
A.
Enterobacteriaceae
p=0.45
Relative abundance (% of total bacteria)
0
20
40
60
80
HIV Negative
HIV Positive
B.
Veillonellaceae
p=0.27
Relative abundance (% of total bacteria)
0
5
10
15
20
25
HIV Negative
HIV Positive
C.
Succinivibrionaceae
p=0.69
Relative abundance (% of total bacteria)
0
10
20
30
40
HIV Negative
HIV Positive
D.
Paraprevotellaceae
p=0.39
Relative abundance (% of total bacteria)
0
5
10
15
HIV Negative
HIV Positive
E.
Erysipelotrichaceae
p=0.06
Relative abundance (% of total bacteria)
0
5
10
15
20
HIV Negative
HIV Positive
F.
Acaligenaceae
p=0.31
Relative abundance (% of total bacteria)
0
5
10
15
HIV Negative
HIV Positive
G.
Bifidobacteriaceae
p=0.05
Relative abundance (% of total bacteria)
0
2
4
6
8
HIV Negative
HIV Positive

Figure S3

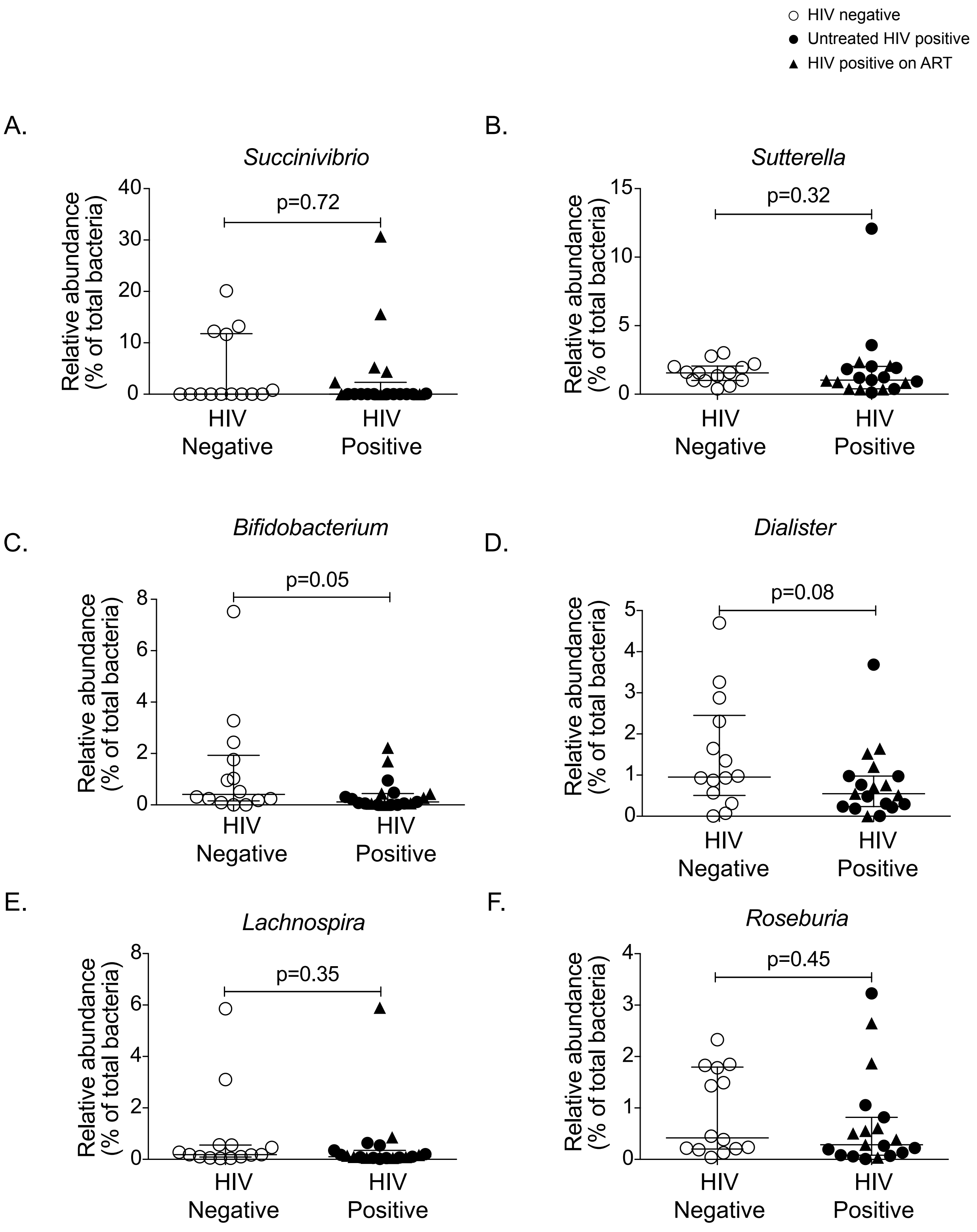

HIV negative
Untreated HIV positive
HIV positive on ART
A.
Succinivibrio
p=0.72
Relative abundance (% of total bacteria)
40
30
20
10
0
HIV Negative
HIV Positive
B.
Sutterella
p=0.32
Relative abundance (% of total bacteria)
15
10
5
0
HIV Negative
HIV Positive
C.
Bifidobacterium
p=0.05
Relative abundance (% of total bacteria)
8
6
4
2
0
HIV Negative
HIV Positive
D.
Dialister
p=0.08
Relative abundance (% of total bacteria)
5
4
3
2
1
0
HIV Negative
HIV Positive
E.
Lachnospira
p=0.35
Relative abundance (% of total bacteria)
8
6
4
2
0
HIV Negative
HIV Positive
F.
Roseburia
p=0.45
Relative abundance (% of total bacteria)
4
3
2
1
0
HIV Negative
HIV Positive

Figure S4

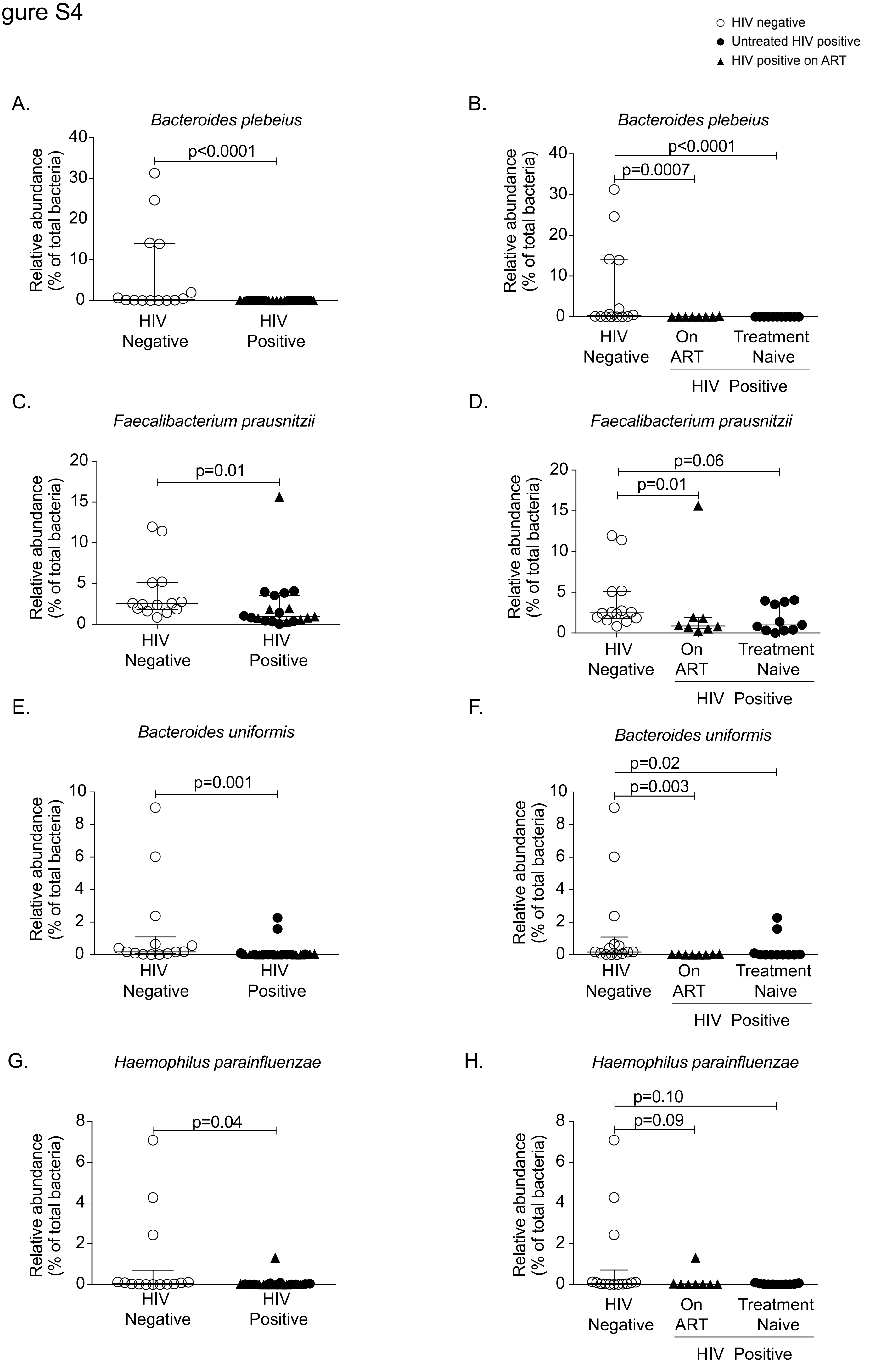
HIV negative
Untreated HIV positive
HIV positive on ART
A.
Bacteroides plebeius
Relative abundance (% of total bacteria)
p<0.0001
HIV Negative
HIV Positive
B.
Bacteroides plebeius
p<0.0001
p=0.0007
HIV Negative
On ART
Treatment Naive
HIV Positive
C.
Faecalibacterium prausnitzii
p=0.01
D.
Faecalibacterium prausnitzii
p=0.06
p=0.01
E.
Bacteroides uniformis
p=0.001
F.
Bacteroides uniformis
p=0.02
p=0.003
G.
Haemophilus parainfluenzae
p=0.04
H.
Haemophilus parainfluenzae
p=0.10
p=0.09

Figure S5

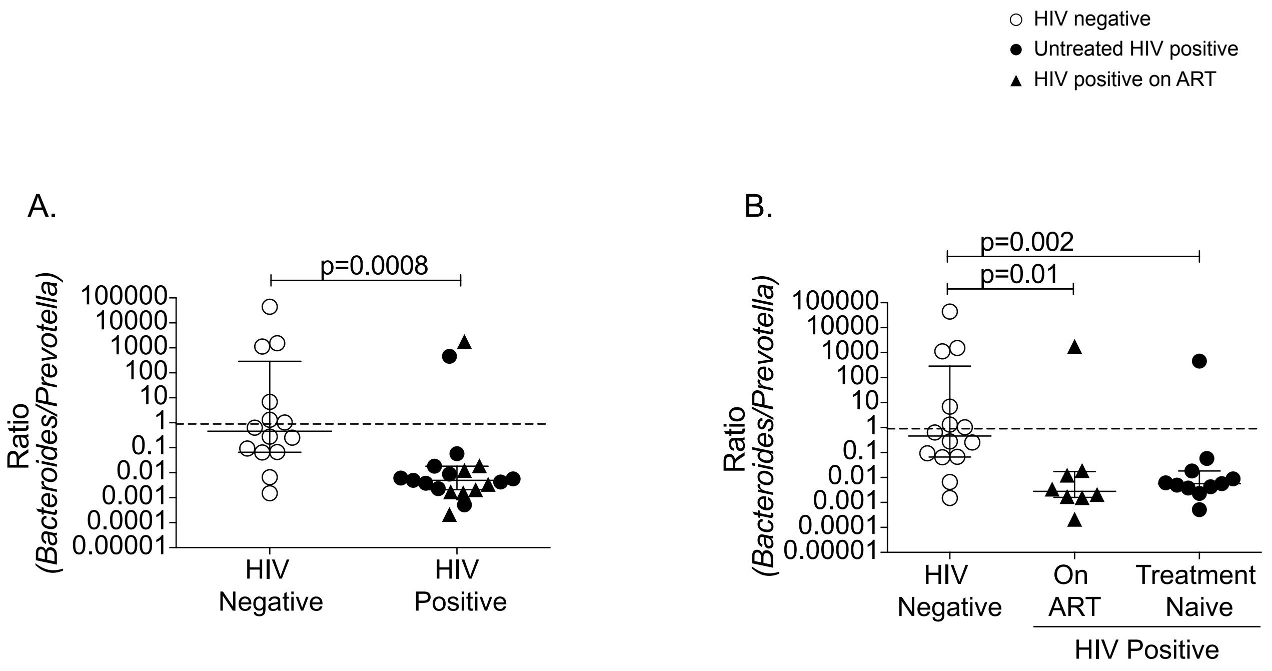

HIV negative
Untreated HIV positive
HIV positive on ART
A.
p=0.0008
Ratio
(Bacteroides/Prevotella)
100000
10000
1000
100
10
1
0.1
0.01
0.001
0.0001
0.00001
HIV
Negative
HIV
Positive
B.
p=0.002
p=0.01
Ratio
(Bacteroides/Prevotella)
100000
10000
1000
100
10
1
0.1
0.01
0.001
0.0001
0.00001
HIV
Negative
On
ART
Treatment
Naive
HIV Positive

Table S1

	HIV Positive	HIV Negative	
	**Median (IQR)**	**Median (IQR)**	**p value**
**Phylum**			
Bacteroidetes	80.03 (56.53 – 87.19)	52.05 (40.66 – 57.70)	0.003
Firmicutes	9.406 (7.388 – 18.51)	36.36 (23.40 – 40.57)	0.002
Actinobacteria	0.195 (0.097 – 0.487)	1.165 (0.409 – 2.888)	0.0007
Spirochaetes	0.001 (0.0 – 0.002)	0.0 (0.0 – 0.0)	0.0007
Cyanobacteria	0.009 (0.004 – 0.037)	0.074 (0.016 – 0.821)	0.03
**Family**			
Prevotellaceae	70.35 (44.84 – 82.96)	28.15 (1.235 – 39.7)	0.0007
Bacteroidaceae	0.33 (0.166 – 0.56)	9.456 (2.151 – 26.02)	0.0008
Ruminococcaceae	3.034 (1.862 – 5.9)	11.31 (4.814 – 19.02	0.005
Lachnospiraceae	1.374 (0.721 – 3.238)	4.429 (1.516 – 7.3)	0.01
Rikenellaceae	0.003 (0.001 – 0.02)	0.33 (0.145 – 1.452)	<0.0001
**Genus**			
Prevotella	70.35 (44.84 – 82.96)	28.15 (1.235 – 39.7)	0.0007
Bacteroides	0.33 (0.166 – 0.56)	9.456 (2.151 – 26.02)	0.0008
Faecalibacterium	0.9185 (0.409 – 3.535)	2.492 (1.776 – 5.116)	0.01
Megasphaera	0.57 (0.002 – 2.784)	0.001 (0.0 – 0.486)	0.02
Ruminococcus	0.136 (0.022 – 0.64)	0.63 (0.244 – 6.088)	0.006
Haemophilus	0.008 (0.003 – 0.03)	0.06 (0.012 – 0.736)	0.04
**Species**			
Prevotella copri	67.45 (40.52 – 76.72)	17.69 (1.121 – 37.44)	0.001

Table S2

	Treatment naive	On ART	HIV Negative
	Median (IQR)	Median (IQR)	Median (IQR)
**Phylum**			
Bacteroidetes	*84.25 (58.48 – 88.83)	74.42 (40.08 – 86.93)	52.05 (40.66 – 57.70)
Firmicutes	*8.56 (5.816 – 12.85)	11.08 (8.593 – 40.91)	36.36 (23.4 – 40.57)
Actinobacteria	*0.134 (0.099 – 0.357)	0.395 (0.075 – 1.528)	1.165 (0.409 – 2.888)
Spirochaetes	*0.0002 (0.0 – 0.002)	* (0.0 – 0.002)	0.0 (0.0 – 0.0)
**Family**			
Prevotellaceae	*81.6 (54.41 – 86.15)	*69.69 (27.9 – 78.88)	28.15 (1.23 – 39.7)
Bacteroidaceae	*0.409 (0.234 – 1.08)	*0.216 (0.122 – 0.526)	9.456 (2.15 – 26.02)
Ruminococcaceae	*4.747 (1.14 – 5.545)	2.643 (1.989 – 18.07)	11.31 (4.814 – 19.02)
Lachnospiraceae	*1.179 (0.524 – 1.678)	1.976 (0.944 – 7.063)	4.429 (1.516 – 7.301)
Rikenellaceae	*0.004 (0.002 – 0.012)	*0.002 (0.0 – 0.03)	0.33 (0.144 – 1.452)
**Genus**			
Prevotella	*81.6 (54.41 – 86.15)	*69.69 (27.9 – 78.88)	28.15 (1.23 – 39.7)
Bacteroides	*0.409 (0.234 – 1.08)	*0.216 (0.122 – 0.526)	9.456 (2.15 – 26.02)
Faecalibacterium	1.001 (0.318 – 3.829)	*0.857 (0.592 – 1.906)	2.492 (1.776 – 5.116)
Megasphaera	*0.573 (0.004 – 5.106)	0.548 (0.0009 – 2.682)	0.001 (0.0 – 0.486)
Ruminococcus	*0.047 (0.014 – 0.136)	0.343 (0.152 – 2.157)	0.633 (0.245 – 3.05)
Haemophilus	0.012 (0.006 – 0.044)	0.008 (0.0 – 0.03)	0.063 (0.012 – 0.737)
**Species**			
Prevotella copri	*68.21 (45.77 – 82.94)	*65.47 (20.01 – 72.85)	17.69 (1.121 – 37.44)

*values that are significantly different from HIV negative controls (p<0.05)

Supplementary figure and table legends:

Table S1: Relative abundance of significantly distinct fecal microbiota between perinatally HIV-infected children and HIV negative controls at phylum, family, genus and species levels.

Table S2: Relative abundance of significantly distinct fecal microbiota across treatment naïve, On ART perinatally HIV-infected children and HIV negative controls at phylum, family, genus and species levels.

Figure S1: Plots representing relative abundance of **A.** Proteobacteria **B.** Cyanobacteria **C**. Lentisphaerae **D**. Fusobacteria and **E**. Tenericutes phyla in HIV negative and HIV positive children. The horizontal line and deviation in the dot plot represents median and interquartile range (IQR), respectively. Mann-Whitney-U-test was performed for statistical analysis. $p<0.05$ was considered to be significant.

Figure S2: Plots representing relative abundance of **A.** Enterobacteriaceae **B.** Veillonellaceae **C**. Succinivibrionaceae **D**. Paraprevotellaceae **E**. Erysipelotrichaceae **F**. Acaligenaceae and **G**. Bifidobacteriaceae families in HIV negative and HIV positive children. The horizontal line and deviation in the dot plot represents median and interquartile range (IQR), respectively. Mann-Whitney-U-test was performed for statistical analysis. $p<0.05$ was considered to be significant.

Figure S3: Plots representing relative abundance of **A.** *Succinivibrio* **B.** *Sutterella* **C**. *Bifidobacterium* **D**. *Dialister* **E**. *Lachnospira* and **F**. *Roseburia* genera in HIV negative and HIV positive children. The horizontal line and deviation in the dot plot represents median and interquartile range (IQR), respectively. Mann-Whitney-U-test was performed for statistical analysis. $p<0.05$ was considered to be significant.

Figure S4: Plots representing relative abundance of **A.** *Bacteroides plebeius* **C.** *Faecalibacterium prausnitzii* **E**. *Bacteroides uniformis* and **G**. *Haemophilus parainfluenzae* species in HIV negative and HIV positive children. Plots representing relative abundance of **B.** *Bacteroides plebeius* **D.** *Faecalibacterium prausnitzii* **F**. *Bacteroides uniformis* and **H**. *Haemophilus parainfluenzae* species across HIV negative controls, On ART and treatment naive HIV positive children. The horizontal line and deviation in the dot plot represents median and interquartile range (IQR), respectively. Mann-Whitney *U*-test and Kruskal-Wallis one-way ANOVA with Dunn's multiple comparison were performed for statistical analysis. $p<0.05$ was considered to be significant.

Figure S5: Plot showing the ratio of the relative abundance of *Bacteroides* to *Prevotella* **A**. between HIV negative and HIV positive children **B**. across HIV negative controls, On ART and treatment naive HIV positive children. Dotted line shows a ratio of 1.0 (relative abundance of *Bacteroides* = relative abundance of *Prevotella*). The horizontal line and deviation in the dot plot represents median and interquartile range (IQR), respectively. Mann-Whitney *U*-test and Kruskal-Wallis one-way ANOVA with Dunn's multiple comparison were performed for statistical analysis
